# Characterizing core microbiota and regulatory functions of the pig gut microbiome

**DOI:** 10.1093/ismejo/wrad037

**Published:** 2024-01-12

**Authors:** Jun Hu, Jianwei Chen, Libao Ma, Qiliang Hou, Yong Zhang, Xiangfeng Kong, Xingguo Huang, Zhonglin Tang, Hong Wei, Xiangru Wang, Xianghua Yan

**Affiliations:** National Key Laboratory of Agricultural Microbiology, Hubei Hongshan Laboratory, Frontiers Science Center for Animal Breeding and Sustainable Production, College of Animal Sciences and Technology, College of Veterinary Medicine, Huazhong Agricultural University, Wuhan, Hubei 430070, China; The Cooperative Innovation Center for Sustainable Pig Production, Wuhan, Hubei 430070, China; Hubei Provincial Engineering Laboratory for Pig Precision Feeding and Feed Safety Technology, Wuhan, Hubei 430070, China; BGI Research, Qingdao, Shandong 266555, China; Laboratory of Genomics and Molecular Biomedicine, Department of Biology, University of Copenhagen, Copenhagen 2100, Denmark; National Key Laboratory of Agricultural Microbiology, Hubei Hongshan Laboratory, Frontiers Science Center for Animal Breeding and Sustainable Production, College of Animal Sciences and Technology, College of Veterinary Medicine, Huazhong Agricultural University, Wuhan, Hubei 430070, China; The Cooperative Innovation Center for Sustainable Pig Production, Wuhan, Hubei 430070, China; Hubei Provincial Engineering Laboratory for Pig Precision Feeding and Feed Safety Technology, Wuhan, Hubei 430070, China; National Key Laboratory of Agricultural Microbiology, Hubei Hongshan Laboratory, Frontiers Science Center for Animal Breeding and Sustainable Production, College of Animal Sciences and Technology, College of Veterinary Medicine, Huazhong Agricultural University, Wuhan, Hubei 430070, China; The Cooperative Innovation Center for Sustainable Pig Production, Wuhan, Hubei 430070, China; Hubei Provincial Engineering Laboratory for Pig Precision Feeding and Feed Safety Technology, Wuhan, Hubei 430070, China; Key Laboratory of Animal Genetics, Breeding and Reproduction in the Plateau Mountainous Region, Ministry of Education, Guizhou University, Guiyang, Guizhou 550025, China; Hunan Provincial Key Laboratory of Animal Nutritional Physiology and Metabolic Process, Institute of Subtropical Agriculture, Chinese Academy of Sciences, Changsha, Hunan 410125, China; College of Animal Science and Technology, Hunan Agriculture University, Changsha, Hunan 410128, China; Shenzhen Branch, Guangdong Laboratory for Lingnan Modern Agriculture, Agricultural Genomics Institute at Shenzhen, Chinese Academy of Agricultural Sciences, Shenzhen 518124, China; National Key Laboratory of Agricultural Microbiology, Hubei Hongshan Laboratory, Frontiers Science Center for Animal Breeding and Sustainable Production, College of Animal Sciences and Technology, College of Veterinary Medicine, Huazhong Agricultural University, Wuhan, Hubei 430070, China; National Key Laboratory of Agricultural Microbiology, Hubei Hongshan Laboratory, Frontiers Science Center for Animal Breeding and Sustainable Production, College of Animal Sciences and Technology, College of Veterinary Medicine, Huazhong Agricultural University, Wuhan, Hubei 430070, China; The Cooperative Innovation Center for Sustainable Pig Production, Wuhan, Hubei 430070, China; National Key Laboratory of Agricultural Microbiology, Hubei Hongshan Laboratory, Frontiers Science Center for Animal Breeding and Sustainable Production, College of Animal Sciences and Technology, College of Veterinary Medicine, Huazhong Agricultural University, Wuhan, Hubei 430070, China; The Cooperative Innovation Center for Sustainable Pig Production, Wuhan, Hubei 430070, China; Hubei Provincial Engineering Laboratory for Pig Precision Feeding and Feed Safety Technology, Wuhan, Hubei 430070, China

**Keywords:** gut microbiome, pig, core gut microbes, gene catalog, germ-free mice

## Abstract

Domestic pigs (*Sus scrofa*) are the leading terrestrial animals used for meat production. The gut microbiota significantly affect host nutrition, metabolism, and immunity. Hence, characterization of the gut microbial structure and function will improve our understanding of gut microbial resources and the mechanisms underlying host–microbe interactions. Here, we investigated the gut microbiomes of seven pig breeds using metagenomics and 16S rRNA gene amplicon sequencing. We established an expanded gut microbial reference catalog comprising 17 020 160 genes and identified 4910 metagenome-assembled genomes. We also analyzed the gut resistome to provide an overview of the profiles of the antimicrobial resistance genes in pigs. By analyzing the relative abundances of microbes, we identified three core-predominant gut microbes (*Phascolarctobacterium succinatutens*, *Prevotella copri*, and *Oscillibacter valericigenes*) in pigs used in this study. Oral administration of the three core-predominant gut microbes significantly increased the organ indexes (including the heart, spleen, and thymus), but decreased the gastrointestinal lengths in germ-free mice. The three core microbes significantly enhanced intestinal epithelial barrier function and altered the intestinal mucosal morphology, as was evident from the increase in crypt depths in the duodenum and ileum. Furthermore, the three core microbes significantly affected several metabolic pathways (such as “steroid hormone biosynthesis,” “primary bile acid biosynthesis,” “phenylalanine, tyrosine and tryptophan biosynthesis,” and “phenylalanine metabolism”) in germ-free mice. These findings provide a panoramic view of the pig gut microbiome and insights into the functional contributions of the core-predominant gut microbes to the host.

## Introduction

The domestic pig (*Sus scrofa*) is a terrestrial animal used for meat production [1] and is widely utilized as a model for biomedical research studies [2–4]. The gut microbiota confer functional contributions to the host, such as degradation of indigestible fibers [5], biosynthesis of vitamins [6], renewal of gut epithelial cells [7], development of colonization resistance against gut pathogens [8], and maturation of the immune system [9]. The gut microbiota are regarded as “organs” in mammals [10]. Manipulation of the gut microbiota composition is considered a promising way of preventing various diseases [11]. Gut microbiota have been linked to several phenotypes, such as diarrhea resistance [12, 13], feed efficiency [14–17], meat quality [18, 19], immune function [20–22], and growth traits [23], in pigs. Understanding the structural and functional landscapes of the gut microbiome will improve our understandings of the gut microbial resources and the mechanisms underlying host–microbe interactions [24].

Core gut microbes can be extensively harbored in the intestinal tract and can dominate the intestinal microbiota for a long time, suggesting stable symbiosis between core gut microbes and the host [25–27]. Thus, core gut microbes may possess powerful metabolic activities, and the probable contribution of core-predominant gut microbes to host health has attracted attention. A previous study identified the core-predominant gut fungus *Kazachstania slooffiae* in pigs and revealed its role in the regulation of gut epithelial glycolysis [28]. Although previous studies have analyzed microbial communities in porcine intestines [29–32], mining of core-predominant gut bacteria is urgently required with considering the differences in pig breeds and growth stages. Thus, mining of the core-predominant gut bacteria in pigs and investigating their functional contributions to the host will shed light on host–microbe interactions.

In this study, we analyzed the structure and function of the fecal microbiome of seven pig breeds and established an expanded gut microbial reference catalog comprising 17 020 160 genes. Three core-predominant gut microbes (*Phascolarctobacterium succinatutens*, *Prevotella copri*, and *Oscillibacter valericigenes*) were identified in pigs using the criterion that these microbial species could be detected in all samples and were the common microbes shared by the top 10 most abundant gut microbial species in both weaned piglets and finishing pigs, as revealed by both metagenomics and 16S rRNA gene amplicon sequencing. *P. copri* and *O. valericigenes* also dominate the human gut microbiota [33, 34]. We systematically evaluated the effects of the three core-predominant gut microbes on host physiology using a germ-free (GF) mouse model. These three core-predominant gut microbes play important roles in maintaining organ indexes, intestinal epithelial barrier function, intestinal mucosal morphology, and host metabolism. Together, these findings provide a panoramic view of pig gut microbiome and suggest the regulatory functions of the core-predominant gut microbes.

## Results

### Expanded pig gut microbial gene catalog constructed using metagenomic analysis

**Figure 1:**
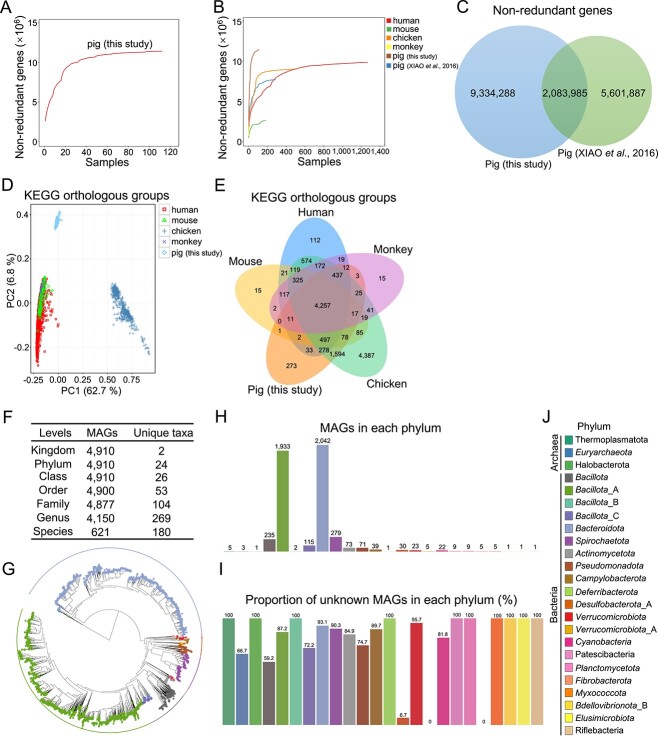
Identification of gut microbial NR genes and MAG profiles in pigs; (A) rarefaction curves based on the gut microbial NR gene profiles in pigs used in this study; (B) rarefaction curves based on gut microbial NR gene profiles in five animal species (human, mouse, chicken, monkey, and pig); (C) Venn diagram of the NR gene profiles in this study and of a previous study examining pigs [29]; (D) PCA of gut microbial KO compositions in five animal species; (E) Venn diagram of gut microbial KOs in five animal species; (F) taxonomic classification of MAGs at different levels; (G) phylogenetic tree of the MAGs; the phyla are marked by different colors; number of MAGs (H) and the proportion of unknown MAGs (I) in each phylum; **(**J**)** phyla are presented in different colors, and this legend was used for G–I.

To systematically analyze the pig gut microbiome, we collected 112 fecal samples from seven representative pig breeds (commercial Duroc × [Landrace × Yorkshire], native Tibetan miniature, native Laiwu, native Shaziling, native Congjiang miniature, native Huanjiang miniature, and native Ningxiang) in China. The animals consisted of 56 weaned piglets and 56 finishing pigs. In total, deep metagenomic sequencing of fecal microbial genomic DNA generated 1432.48 Gbp of clean high-quality data. An average of 12.79 Gbp clean data were obtained per sample. Rarefaction analysis revealed that almost all microbial genes in the samples were captured (Fig. 1A).

A total of 11 418 273 nonredundant (NR) gut microbial genes were identified using de novo assembly (Fig. 1A), as described previously [28], with the average N50 contig length of the NR genes being ~1658.7 bp. The rarefaction curves based on gene profiles allowed the comparison of the gut microbial NR genes among five animal species (human [35], mouse [36], chicken [37], monkey [38], and pig) (Fig. 1B). A total of 2 083 985 common NR genes were identified in both ours and a previous pig gut microbial gene catalog [29], and 9 334 288 unique NR genes were identified in our pig gut microbial gene catalog (Fig. 1C), suggesting a rich gut microbial gene resource for native pig breeds in China. By integrating our de novo-assembled gene catalog with the NR genes in the pig gut microbiome that were reported previously [29], we constructed a new expanded pig gut microbial gene catalog containing 17 020 160 (17.02 million) NR genes. We also showed the numbers of NR gut microbial genes quantified (Fig. S1A) and the Shannon’s diversity of the NR gut microbial genes quantified (Fig. S1B) in 112 pigs from seven pig breeds, respectively. These results suggested small individual differences within group. The results also indicated that the number of NR gut microbial genes quantified in finishing pigs was significantly larger than that in weaned piglets (Fig. S1A). Shannon’s diversity indicated that the diversity of NR gut microbial genes quantified in finishing pigs was significantly higher than that in weaned piglets (Fig. S1B). Principal coordinates analysis (PCoA) of the NR gut microbial genes further suggested an obvious distinction among pig breeds and a small individual difference within the group (Fig. S1C). The scatter plot derived from principal component analysis (PCA) showed the gut microbial compositions of Kyoto Encyclopedia of Genes and Genomes (KEGG) orthologous groups (KOs) in five animal species (human, mouse, chicken, monkey, and pig) (Fig. 1D). We identified a total of 4257 core functional KOs, indicating a large overlap of gut microbial functional KOs among these five animal species (Fig. 1E). Our data also identified 273 unique functional gut microbial KOs in pigs (Fig. 1E). We constructed a new expanded pig gut microbial gene catalog containing 17.02 million NR genes and compared the gut microbial KOs of the five animal species.

By reconstructing prokaryotic genomes from metagenomic data, 4910 metagenome-assembled genomes (MAGs) were obtained based on the quality control metrics (>50% completeness and < 5% contamination) (Fig. 1F). Among the 11 418 273 NR gut microbial genes identified in this study, a total of 9 141 561 NR genes matched the MAGs, and the coverage rate was ~80.1%. Three phyla belonged to archaea and 21 phyla belonged to bacteria. The 4877 MAGs (99.3% of the total MAGs) were annotated into 104 families, and 4150 MAGs (84.5% of the total MAGs) were assigned to 269 genera. A total of 621 MAGs were assigned to 180 known species, suggesting the potential for exploring virgin gut microbial species in pigs (Fig. 1F). The phylogenetic tree of MAGs showed that 2285 MAGs (46.5% of total MAGs) belonged to *Bacillota* and 2042 MAGs (41.6% of total MAGs) belonged to *Bacteroidota*, suggesting that *Bacillota* and *Bacteroidota* were two major phyla in pig gut microbiota (Fig. 1G and H). The results showed that all MAGs belonging to two phyla (*Verrucomicrobiota*_A and *Fibrobacterota*) could be classified at the species level and were defined as known MAGs (Fig. 1I and J). However, none of the MAGs belonging to Thermoplasmatota, Halobacterota, *Bacillota*_B, *Deferribacterota*, Patescibacteria, *Planctomycetota*, *Myxococcota*, *Bdellovibrionota*_B, *Elusimicrobiota*, and Riflebacteria could be assigned to any species and were defined as unknown MAGs (Fig. 1I and J).

### Functional landscape of the pig gut microbiome revealed by metagenomics

**Figure 2:**
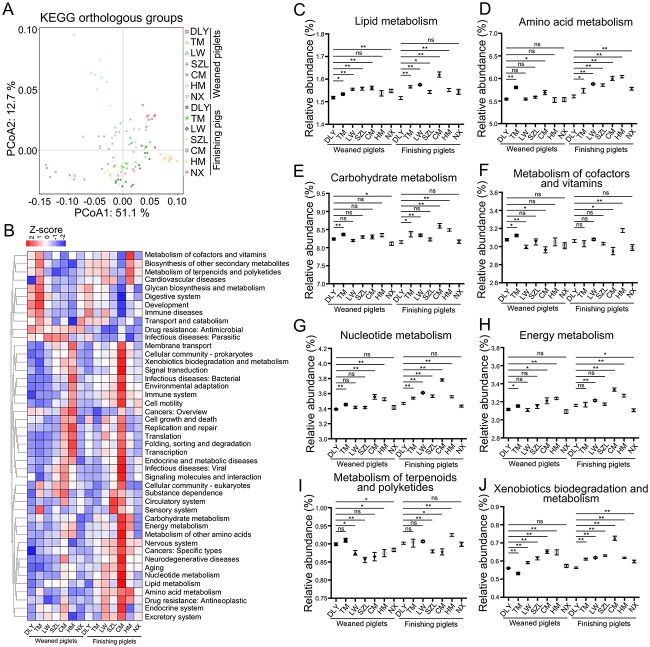
Analysis of gut microbial KEGG metabolism profiles; (A) scatter plot from PCoA of gut microbial KO compositions in pigs (DLY, Duroc × [landrace × Yorkshire]; TM, Tibetan miniature; LW, Laiwu; SZL, Shaziling; CM, Congjiang miniature; HM, Huanjiang miniature; NX, Ningxiang); (B) heatmap analysis of KEGG pathways; comparative analyses of the relative abundances of gut microbial genes involved in KEGG pathways, including lipid metabolism (C), amino acid metabolism (D), carbohydrate metabolism (E), metabolism of cofactors and vitamins (F), nucleotide metabolism (G), energy metabolism (H), metabolism of terpenoids and polyketides (I), and xenobiotics biodegradation and metabolism (J); data are presented as mean ± SEM (*n* = 8) and were evaluated by the Kruskal–Wallis test; ^*^^*^P < 0.01, ^*^P < 0.05; ns, not significant.

The scatter plot derived from the PCoA of metagenomics data revealed grouping of the composition of gut microbial KOs according to the pig breed and difference in the composition of gut microbial KOs among pig breeds (Fig. 2A). The heatmap further revealed differences in relative abundances of gut microbial KEGG metabolism-related genes among pig breeds (Fig. 2B). Native pigs had significantly higher relative abundances of several KEGG metabolic pathways-related genes, such as those related to lipid metabolism (Fig. 2C), biosynthesis of unsaturated fatty acids (Fig. S2A), and secondary bile acid biosynthesis (Fig. S2B), amino acid metabolism (Fig. 2D), tryptophan metabolism (Fig. S2C), and phenylalanine metabolism (Fig. S2D), carbohydrate metabolism (Fig. 2E), propanoate metabolism (Fig. S2E), and butanoate metabolism (Fig. S2F), than commercial pigs (Duroc × [Landrace × Yorkshire]). However, the metabolism of cofactors and vitamins was not significantly higher in native pigs (excluding Huanjiang miniature) than in commercial pigs (Duroc × [Landrace × Yorkshire]) (Fig. 2F). Furthermore, native pigs had significantly higher relative abundances of several KEGG metabolic pathways-related genes, such as those involved in nucleotide metabolism (Fig. 2G), purine metabolism (Fig. S2G), pyrimidine metabolism (Fig. S2H), energy metabolism (Fig. 2H), methane metabolism (Fig. S2I), and nitrogen metabolism (Fig. S2J), than commercial pigs (Duroc × [Landrace × Yorkshire]). However, the metabolism of terpenoids and polyketides was not significantly higher in native pigs (excluding Huanjiang miniature) than in commercial pigs (Duroc × [Landrace × Yorkshire]) (Fig. 2I). Native pigs had significantly higher relative abundances of several KEGG metabolic pathways-related genes, such as those related to xenobiotics biodegradation and metabolism (Fig. 2J), benzoate degradation (Fig. S2K), and dioxin degradation (Fig. S2L), than commercial pigs (Duroc × [Landrace × Yorkshire]). Together, these data revealed that the gut microbiome of native pigs may have significantly stronger capacities for nutrient (such as lipid, amino acid, carbohydrate, and nucleotide), xenobiotics, and energy metabolism than those of commercial pigs.

### Characterization of the pig gut resistome using metagenomics

**Figure 3:**
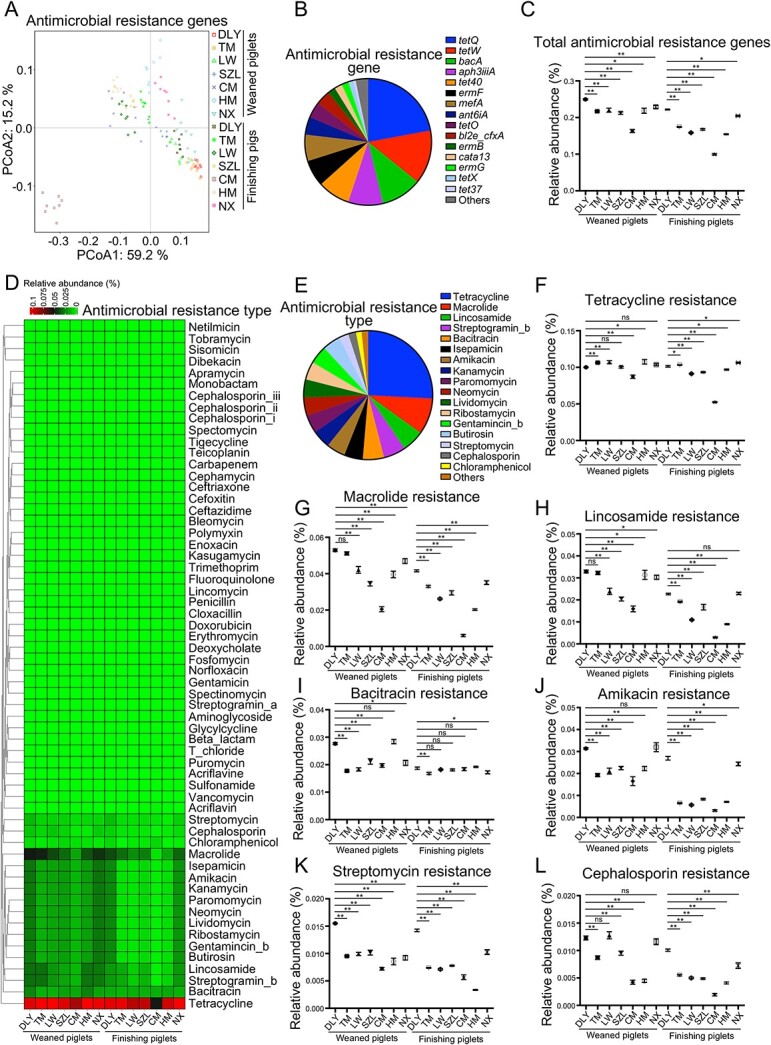
Analysis of gut microbial ARG profiles; (A) scatter plot from PCoA of gut microbial ARG compositions in pig; (B) average proportions of gut microbial ARGs; (C) comparative analysis of relative abundances of total ARGs; (D) heatmap analysis of gut microbial antimicrobial resistance types; (E) average proportions of gut microbial antimicrobial resistance types; comparative analyses of the relative abundances of the antimicrobial resistance types, including tetracycline (F), macrolide (G), lincosamide (H), bacitracin (I), amikacin (J), streptomycin (K), and cephalosporin (L) resistances; data are presented as mean ± SEM (*n* = 8) and were evaluated by the Kruskal–Wallis test in C and F–L; ^*^^*^P < 0.01, ^*^P < 0.05; ns, not significant.

Considering that the dissemination of antimicrobial-resistant microbes has become a serious problem in animal husbandry [39], we analyzed the antimicrobial resistance gene (ARG) profiles in the pig gut microbiome. The results of PCoA revealed a difference in the composition of gut microbial ARGs among the pig breeds (Fig. 3A). A total of 126 gut microbial ARGs were identified in the pigs used in this study. Of these, several ARGs (such as *tetQ*, *tetW*, *bacA*, *aph3iiiA*, *tet40*, *ermF*, and *mefA*) were dominated in the pig gut microbiome (Fig. 3B). The relative abundance of total ARGs was significantly highest in commercial Duroc × [Landrace × Yorkshire] weaned piglets among that in all the weaned piglets, and the relative abundance of total ARGs was significantly highest in commercial Duroc × [Landrace × Yorkshire] finishing pigs among those in all the finishing pigs (Fig. 3C), suggesting a large risk of ARG dissemination in commercial pigs. However, the relative abundance of total ARGs was lowest in native Congjiang miniature weaned piglets among those in all the weaned piglets, and the relative abundance of total ARGs was lowest in native Congjiang miniature finishing pigs among those in all the finishing pigs, suggesting low risk of ARG dissemination in native Congjiang miniature pigs (Fig. 3C). Spearman’s correlation analysis was used to identify potential gut microbial species that may be associated with ARGs. The heatmap indicates that *Escherichia coli* correlated significantly and positively with several ARGs, including *tetA*, *tetB*, *tetC*, *acrB*, *mdtF*, *acrA*, *macB*, *arnA*, *emrD*, *cml_E1*, *bl1_EC*, *bcR*, *mdtH*, *ksgA*, *mdtN*, *mdtO*, *mdfA*, *mdtL*, *mdtG*, *mdtP*, *mdtE*, *mdtK*, *qnrS*, *mdtM*, and *tolC* (Fig. S3).

We analyzed the antimicrobial resistance phenotypes of the pig gut microbiome. Sixty antimicrobial resistance phenotypes were classified, and differences in the relative abundances of antimicrobial resistance phenotypes among pig breeds were identified (Fig. 3D). Among these, several antimicrobial resistance phenotypes (such as resistance to tetracycline, macrolide, lincosamide, streptogramin_b, bacitracin, isepamicin, amikacin, kanamycin, paromomycin, neomycin, lividomycin, ribostamycin, gentamincin_b, butirosin, streptomycin, cephalosporin, and chloramphenicol) dominated the antimicrobial resistance landscape of the pig gut microbiome (Fig. 3E). A detailed analysis of antimicrobial resistance phenotypes (such as resistance to tetracycline, macrolide, lincosamide, bacitracin, amikacin, streptomycin, and cephalosporin) further suggested a significantly higher risk of ARG spreading in commercial Duroc × [Landrace × Yorkshire] pigs than in native Chinese pigs (Fig. 3F–L). Spearman’s correlation analysis was used to identify potential gut microbial species that may be associated with antimicrobial resistance phenotypes. *E. coli* showed significant positive correlation with several antimicrobial resistance phenotypes, including those against glycylcycline, aminoglycoside, beta_lactam, norfloxacin, acriflavin, acriflavine, deoxycholate, fosfomycin, puromycin, t_chloride, kasugamycin, polymyxin, enoxacin, doxorubicin, and erythromycin (Fig. S4). Together, these findings provide a primary overview of ARGs in the pig gut microbiome and suggest that commercial pigs are at significantly higher risk of spreading gut microbial ARGs than native Chinese pigs, and a potential correlation between *E. coli* and antimicrobial resistance.

### Landscape of the pig gut bacterial communities revealed by 16S rRNA gene amplicon sequencing

**Figure 4:**
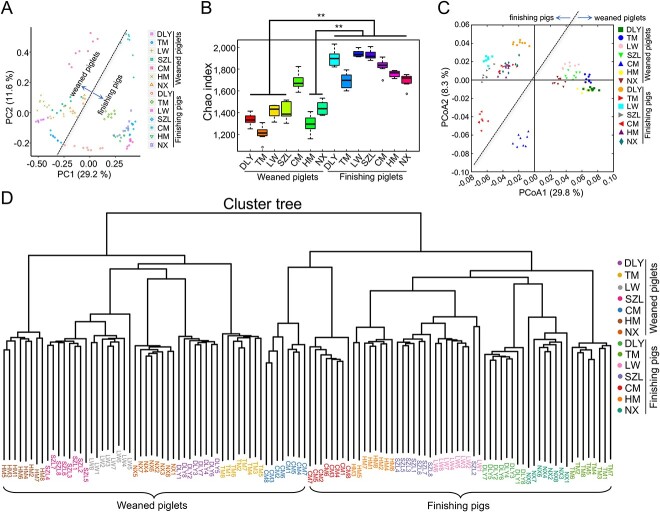
Pig gut bacterial community revealed using 16S rRNA gene sequencing; (A) scatter diagram of OTUs from PCA; (B) Chao index analysis; (C) scatter diagram for PCoA of bacterial communities based on UniFrac distance; (D) cluster tree analysis; data are presented as mean ± SEM (*n* = 8) and were evaluated by the Kruskal–Wallis test in B; ^*^^*^P < 0.01.

Considering that the proportion of gut bacteria is highest among the gut microbes in pigs [29, 30], we also performed 16S rRNA gene sequencing to analyze the bacterial communities in pigs. The use of two methods (including 16S rRNA gene amplicon sequencing and metagenomics) contributes to obtaining more precise results of bacterial communities. The sequencing depth was sufficient to detect the microbial species, as shown in rarefaction curves (Fig. S5A). The results of PCA revealed a difference in the operational taxonomic unit (OTU) composition between weaned piglets and finishing pigs (Fig. 4A). The finishing pigs showed significantly higher gut bacterial species richness than the weaned piglets (except for Congjiang miniature) (Fig. 4B). The results of the PCoA based on UniFrac distance and cluster tree revealed a difference in the bacterial community between weaned piglets and finishing pigs, and a difference in bacterial community among pig breeds (Fig. 4C and D). The heatmap further indicated that several core genera (such as *Treponema*, *Phascolarctobacterium*, *Oscillibacter*, *Bacteroides*, and *Prevotella*) dominated the porcine bacterial community (Fig. S5B).

### Screening of core microbial species predominant in the pig gut microbiota

**Figure 5:**
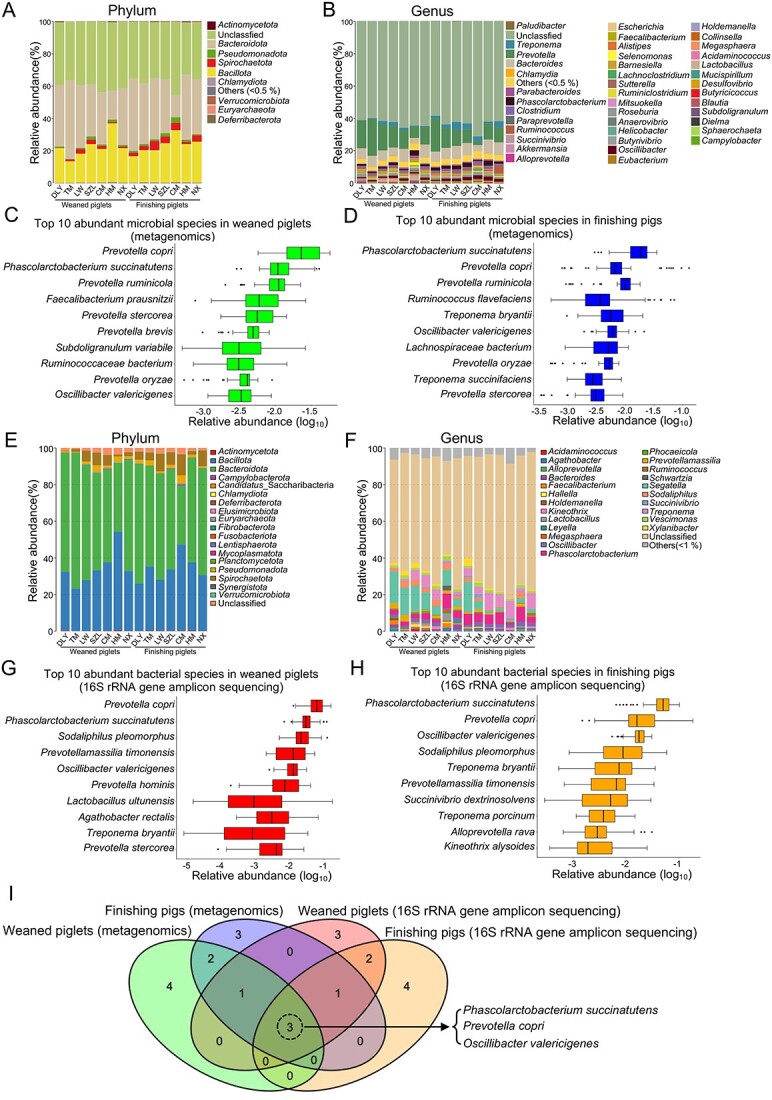
Screening of core-predominant gut microbial species in pigs; microbial taxonomic compositions at the phylum (A) and genus (B) levels, as revealed by metagenomics, respectively; top 10 most abundant microbial species in 56 weaned piglets (C) and 56 finishing pigs (D), as revealed by metagenomics, respectively; gut bacterial taxonomic compositions at phylum (E) and genus (F) levels, as revealed by 16S rRNA gene sequencing, respectively; top 10 most abundant bacterial species in 56 weaned piglets (G) and 56 finishing pigs (H), as revealed by 16S rRNA gene sequencing, respectively; (I) Venn diagram of the core gut microbial species that were predominant in pigs based on the four results of screening the top 10 most abundant microbial species in C, D, G, and H.

We screened the core-predominant gut microbes in pigs by combining results from metagenomics and 16S rRNA gene sequencing, as well as by considering the occurrence of microbes, the relative abundances of microbes, and “persistent” core microbes measured across timescales, as recommended previously [27]. Metagenomic analysis indicated that microbes (including bacteria, fungi, viruses, and archaea) were present in the intestinal tract of pigs, and that the proportion of bacteria was the highest (Fig. S6A). Differences in the microbial taxonomic compositions were observed among pig breeds at several levels (including phylum [Fig. 5A], class [Fig. S6B], order [Fig. S6C], family [Fig. S6D], and genus [Fig. 5B]) in the metagenomics analysis. We further screened the top 10 most abundant microbial species that dominated the weaned piglets (Fig. 5C) and finishing pigs (Fig. 5D) by metagenomics, respectively. The top 10 most abundant gut microbial species belonged to bacteria. Based on the results of the 16S rRNA gene sequencing, we also analyzed the bacterial taxonomic compositions (including phylum [Fig. 5E], class [Fig. S6E], order [Fig. S6F], family [Fig. S6G], and genus [Fig. 5F]) among the different pig breeds. The top 10 most abundant bacterial species in weaned piglets (Fig. 5G) and finishing pigs (Fig. 5H) were identified using 16S rRNA gene sequencing. The top 10 most abundant bacterial species were observed in all samples (Fig. 5C, D, G, and H). The Venn diagram (Fig. 5I) showed that three core-predominant bacterial species (including *P. succinatutens*, *P. copri*, and *O. valericigenes*) were shared by the top 10 most abundant gut microbial species in the four histograms (Fig. 5C, D, G, and H) used for the screening of core gut microbial species. Thus, we determined that these three core gut microbial species (*P. succinatutens*, *P. copri*, and *O. valericigenes*) were predominant in the intestinal tracts of pigs.

### Functional contributions of core-predominant gut microbial species to the host

**Figure 6:**
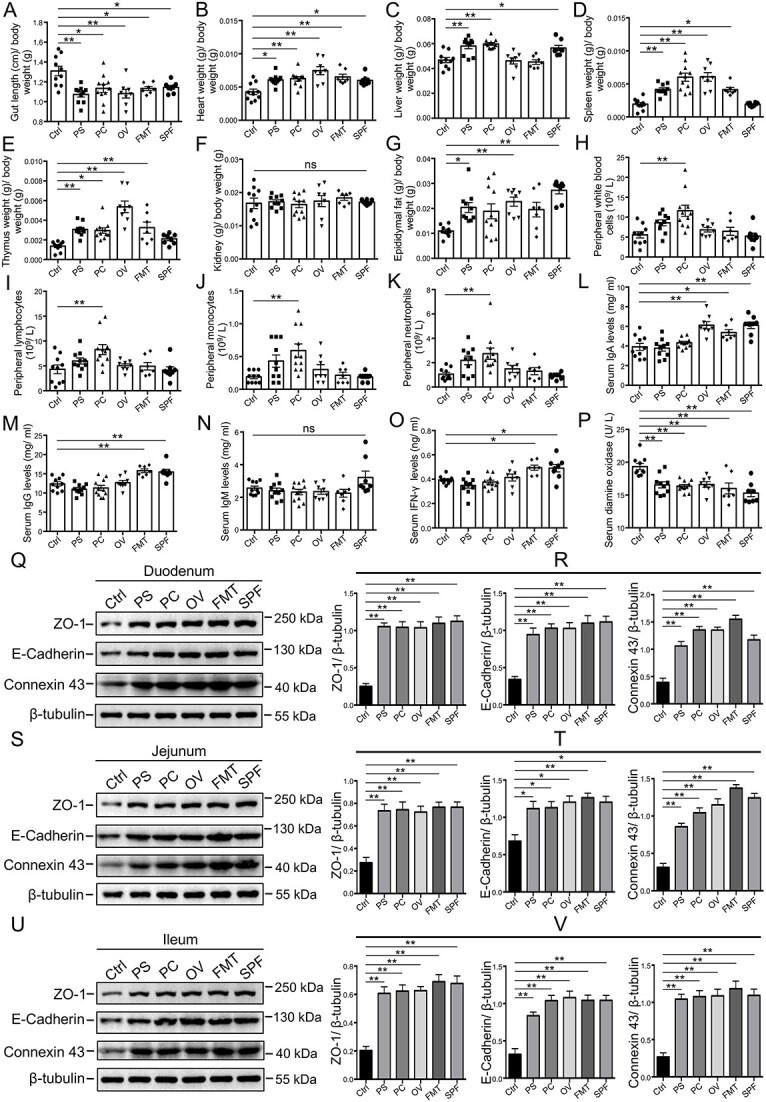
Effects of core-predominant microbes on organ index, blood routine parameters, serum immunoglobulin levels, and intestinal barrier function in GF mice; (A) proportion of gut length to body weight in mice (Ctrl, control; PS, *P. succinatutens*; PC, *P. copri*; OV, *O. valericigenes*;); organ indexes for the heart (B), liver (C), spleen (D), thymus (E), and kidney (F) in mice; (G) proportion of epididymal fat to body weight in mice; numbers of routine blood cells, including white blood cells (H), lymphocytes (I), monocytes (J), and neutrophils (K); levels of serum IgA (L), IgG (M), IgM (N), and IFN-γ (O) in mice; (P) activities of serum diamine oxidase; (Q–V) western blot of ZO-1, E-cadherin, connexin 43, and β-tubulin in duodenal (Q), jejunal (S), and ileal (U) epithelium of mice; quantitation of ZO-1, E-cadherin, and connexin 43 levels normalized to β-tubulin levels in duodenal (R), jejunal (T), and ileal (V) epithelium of mice; data are presented as mean ± SEM and were evaluated by one-way ANOVA; *n* = 10 (ctrl and PS), *n* = 11 (PC), *n* = 8 (OV and SPF), and *n* = 7 (FMT) for A-P; *n* = 3 for R, T, and V; ^*^^*^P < 0.01, ^*^P < 0.05; ns, not significant.

**Figure 7:**
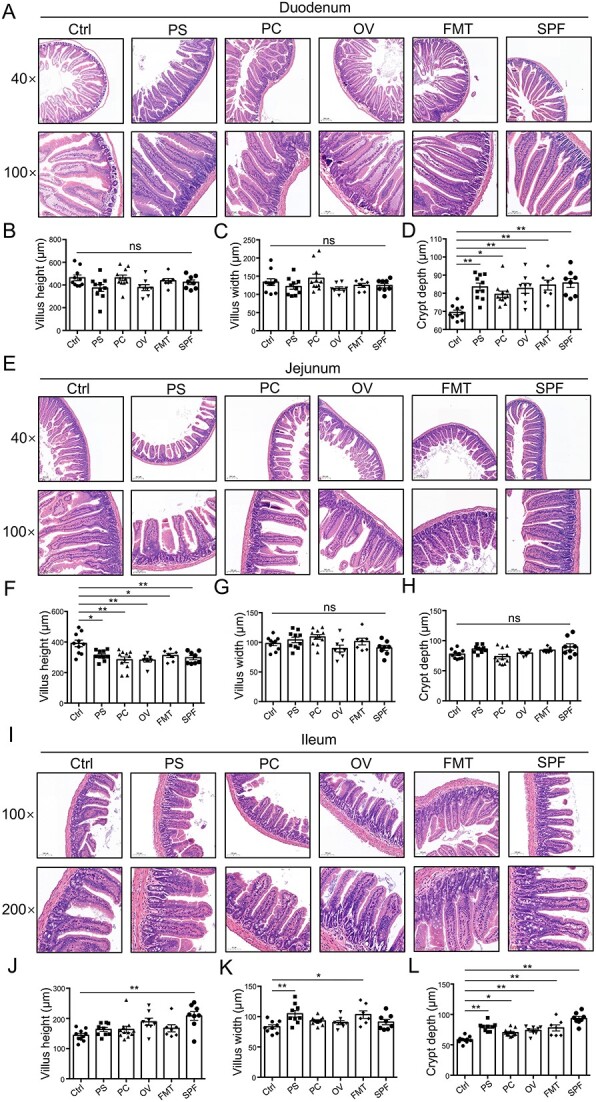
Effects of core-predominant microbes on gut histological morphology in GF mice; (A–D) duodenal histological morphology; the representative images showing histological morphology are presented in A; statistical analysis of villus height (B), villus width (C), and crypt depth (D); (E–H) Jejunal histological morphology; the representative images of histological morphology are presented in E; statistical analysis of villus height (F), villus width (G), and crypt depth (H); (I–L) ileal histological morphology; the representative images showing histological morphology are presented in I; statistical analysis of villus height (J), villus width (K), and crypt depth (L); data are presented as mean ± SEM and were assessed by one-way ANOVA; *n* = 10 (Ctrl and PS), *n* = 11 (PC), *n* = 8 (OV and SPF), *n* = 7 (FMT). ^*^^*^P < 0.01, ^*^P < 0.05; ns, not significant.

We evaluated the roles of three core-predominant gut microbes (*P. succinatutens*, *P. copri*, and *O. valericigenes*) isolated from feces of pigs in the host using a GF mouse model. Our data showed that these three core gut microbes and fecal microbiota transplantation (FMT) significantly decreased the gut lengths (Fig. 6A) and increased organ indexes (including the heart, spleen, and thymus) in GF mice (Fig. 6B–F). Oral administration of *P. succinatutens* and *O. valericigenes* significantly increased epididymal fat weight (Fig. 6G). Oral administration of *P. copri* significantly increased the number of blood immune cells (white blood cells, lymphocytes, monocytes, and neutrophils) in GF mice (Fig. 6H–K). Serum IgA levels were significantly increased by *O. valericigenes* administration, FMT, and natural microbial colonization (Fig. 6L). The levels of serum IgG and interferon-γ (IFN-γ) were significantly increased by both FMT and natural microbial colonization, but were not altered by the three core gut microbes (Fig. 6M–O). The results showed that oral administration of these three core gut microbes significantly decreased the activities of serum diamine oxidase (Fig. 6P) and increased the expression of intestinal epithelial Zonula Occludens (ZO)-1 (a tight junction protein), E-cadherin (an adhesion junction protein), and connexin 43 (a gap junction protein) (Fig. 6Q–V). These data indicated that these three core gut microbes can enhance intestinal epithelial barrier function, suggesting the potential beneficial roles of three core gut microbes in host health. The three core gut microbes significantly altered intestinal mucosal morphology, as shown by the increase in crypt depths in both the duodenum and ileum (Fig. 7). All three core gut microbes (*P. succinatutens*, *P. copri*, and *O. valericigenes*) were identified in the feces of recipient GF mice after FMT (Fig. S7A). *O. valericigenes* was also identified in the feces of specific pathogen-free (SPF) mice (natural microbial colonization) (Fig. S7B).

**Figure 8:**
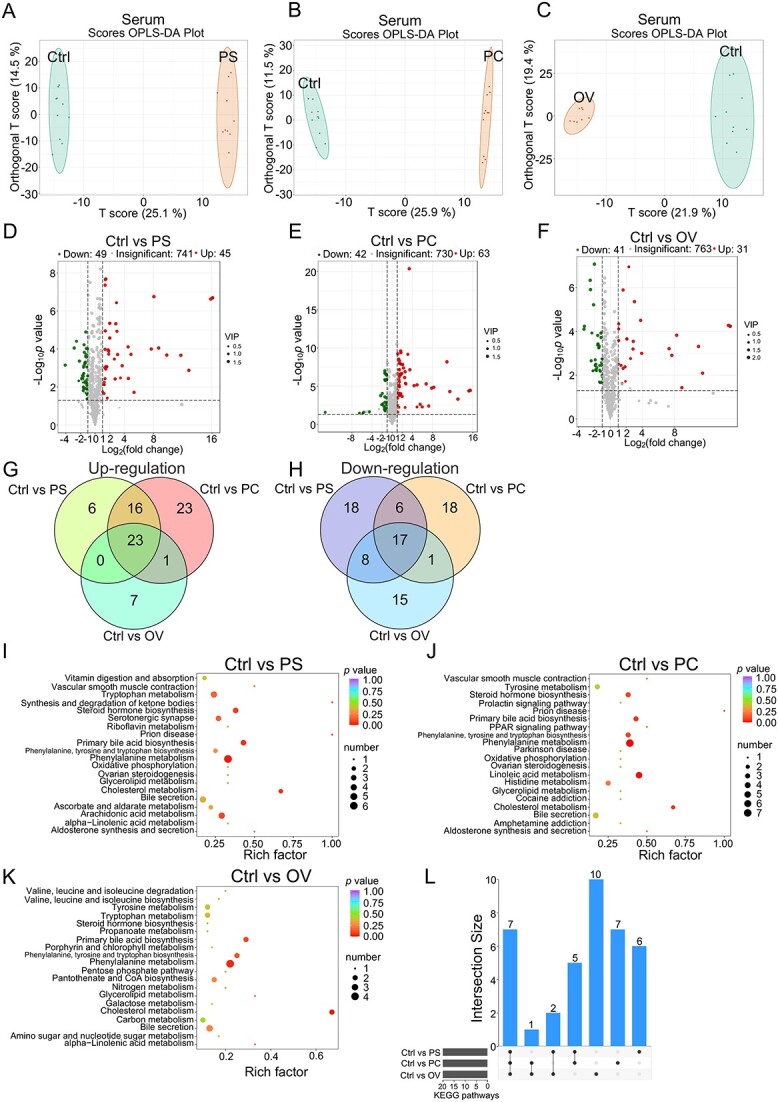
Effects of core-predominant microbes on the composition of serum metabolites in GF mice; OPLS-DA for serum metabolites in mice from groups (Ctrl and PS) (A), groups (Ctrl and PC) (B), and groups (Ctrl and OV) (C); volcano plot analysis of serum metabolites in mice from groups (Ctrl and PS) (D), groups (Ctrl and PC) (E), and groups (Ctrl and OV) (F); (G) Venn diagram analysis of serum metabolites that are upregulated by treatments with the three core-predominant microbes, respectively; (H) Venn diagram analysis of serum metabolites that are downregulated by treatments with the three core-predominant microbes, respectively; the KEGG enrichment analyses of the serum metabolites altered by the treatments with *P. succinatutens* (I), *P. copri* (J), and *O. valericigenes* (K), respectively; (L) UpSet plot comparing the KEGG pathways enriched with the serum differentially metabolites induced by the treatments with *P. succinatutens*, *P. copri*, and *O. valericigenes*, respectively; the differentially abundant metabolites were assessed based on VIP ≥1 from the OPLS-DA, absolute Log2 (fold change) ≥1, and *P* value <0.05.

We investigated the effects of the three core-predominant gut microbes (*P. succinatutens*, *P. copri*, and *O. valericigenes*) on the composition of metabolites in the serum and feces. The orthogonal partial least squares discrimination analysis (OPLS-DA) plot (Fig. 8A–C) and volcano plot (Fig. 8D–F) showed that the composition of serum metabolites in GF mice was altered by *P. succinatutens*, *P. copri*, and *O. valericigenes*, respectively. Metabolomics identified 23 common serum metabolites whose levels were significantly increased by *P. succinatutens*, *P. copri*, and *O. valericigenes*, collectively (Fig. 8G). However, metabolomics identified 17 common serum metabolites whose levels were significantly decreased by *P. succinatutens*, *P. copri*, and *O. valericigenes*, collectively (Fig. 8H). Enrichment analysis of KEGG pathways indicated that seven common metabolic pathways, including “steroid hormone biosynthesis,” “primary bile acid biosynthesis,” “phenylalanine, tyrosine and tryptophan biosynthesis,” “phenylalanine metabolism,” “glycerolipid metabolism,” “cholesterol metabolism,” and “bile secretion,” were significantly enriched with those differentially serum metabolites induced by the three core-predominant gut microbes, respectively (Fig. 8I–L). Our results also demonstrated that the composition of fecal metabolites in GF mice was altered by the treatment with three core gut microbes, as shown in the OPLS-DA plot (Fig. S8A–C) and volcano plots (Fig. S8D–F). Metabolomics analysis identified 234 common fecal metabolites whose levels were significantly increased by *P. succinatutens*, *P. copri*, and *O. valericigenes*, collectively (Fig. S8G). However, metabolomics analysis identified 79 common fecal metabolites whose levels were significantly decreased by *P. succinatutens*, *P. copri*, and *O. valericigenes*, collectively (Fig. S8H). Enrichment analysis of KEGG pathways indicated that five common metabolic pathways, including “vitamin digestion and absorption,” “vitamin B6 metabolism,” “tryptophan metabolism,” “phenylalanine metabolism,” and “metabolic pathways” were significantly enriched with those differentially fecal metabolites induced by the three core-predominant gut microbes, respectively (Fig. S8I–L). These data indicated that the phenylalanine metabolism is a common pathway that was significantly enriched with those differentially serum and fecal metabolites. The results of complete genome sequences also indicated that several KEGG metabolic pathways (such as phenylalanine, vitamin B6, and tryptophan metabolism) could be predicted in the three core-predominant gut microbes based on genome annotations (Fig. S9), and these results were consistent with the fecal metabolomics data. Together, these findings revealed that the three core-predominant gut microbes play important roles in maintaining host organ indexes, intestinal epithelial barrier function, intestinal mucosal morphology, and nutrition metabolism.

## Discussion

Pigs, one of the leading terrestrial animals used in global meat production [1], have been widely used as biomedical models to investigate the physiological functions of humans [2, 3]. Hence, analysis of the gut microbiome in pigs and identification of the gut microbes that confer functional contributions to the host are critical. In this study, we constructed an expanded gut microbial reference gene catalog for pigs using metagenomics based on 112 fecal samples from seven pig breeds in China. The number of NR genes (11.4 million) de novo-assembled in our study was ~1.5-fold more than the number of NR genes (7.7 million) identified previously, although the number of our sequenced samples was considerably lesser than that reported previously [29]. These results suggested that native Chinese pig breeds are rich in gut microbial gene resources. By integrating our de novo-assembled gene catalog with the NR genes in the pig gut microbiome reported previously [29], we constructed a new expanded pig gut microbial gene catalog containing 17 020 160 NR genes (17.02 million). This number of NR genes was approximately equal to the number of NR genes (17.23 million) reported in a recent study of the pig gut microbiome [30]. The identified porcine gut microbial genes provide fundamental reference resources for gut microbiome-associated studies involving metagenomics, metatranscriptomics, and metaproteomics.

In general, native Chinese pigs (such as Laiwu) have a significantly higher body fat mass than commercial pigs (such as the classical lean Duroc × [Landrace × Yorkshire] breed) [40–42]. Our data demonstrated that the gut microbial lipid metabolic capacity of native pigs was significantly stronger than that of commercial pigs, further suggesting that host fat synthesis may be mediated by gut microbial lipid metabolic activity. Secondary bile acid biosynthesis, which depends on the roles of gut microbiota [43], plays important roles in promoting dietary digestion and eliminating gut pathogens [44]. Our data demonstrated that the relative abundance of secondary bile acid biosynthesis-related genes in native pigs was significantly higher than that in commercial pigs, further suggesting that native pigs may digest complex food better and show greater disease resistance than commercial pigs [12, 45]. Considering that the gut microbiota are essential for the digestion of crude fiber in intestinal tract [46], the carbohydrate metabolism capacity of the gut microbiota may affect roughage resistance in pigs. Our results indicated that the relative abundance of genes related to carbohydrate metabolism (such as propanoate and butanoate) in native pigs was significantly higher than that in commercial pigs, further supporting the previous conclusion that native pigs have significantly higher roughage resistance than commercial pigs [45, 47]. Considering that pigs of different breeds were not fed same diet and exposed to identical environments in this study, the differences in the gut microbiome among the seven pig breeds may be attributed to several factors, such as breeds, diet, and environment.

Our results also showed that commercial pigs had the significantly highest relative abundance of total gut microbial ARGs among pigs (including six native pig breeds and one commercial pig breed), indicating a large risk for the spread of antimicrobial-resistant gut microbes in commercial pigs. This spread of antimicrobial-resistant microbes may be caused by the overuse of antibiotics in commercial pig farms [39, 48], because commercial breeds have significantly lower disease resistance than native breeds [12, 45]. Consistent with the results of a previous study [49], our Spearman’s correlation analysis suggested that *E. coli* showed the highest positive correlation with several ARGs. Previous studies have also shown the prevalence of antimicrobial-resistant *E. coli* on pig farms by analyzing the isolates of *E. coli* [50–52]. These findings underscore the enormity of the risk of antimicrobial-resistant *E. coli* dissemination in the livestock industry. These gut microbial gene resources in pigs will provide insights regarding the risk of antimicrobial-resistant gut microbes dissemination and potential relationships between the gut microbiota and host metabolism. Thus, analysis of the gut microbial structure and function in pigs is important.

The core gut microbes are predominant in the gut microbiota, suggesting a stable symbiosis between the host and core gut microbes [25]. Thus, the core gut microbes may play vital regulatory roles in host physiology and growth, and may be promising targets for mediating host metabolism. In this study, we identified three core-predominant gut microbes (*P. succinatutens*, *P. copri*, and *O. valericigenes*) in pigs. *P. copri* and *O. valericigenes* are also predominant in the human intestinal microbiota [33, 34]. Previous studies have demonstrated that *P. copri* can aggravate glucose intolerance and induce insulin resistance [53], whereas it is positively associated with dietary fiber-induced improvements in glucose homeostasis [54]. Recent studies have suggested potential links between *P. copri* and susceptibility to rheumatoid arthritis [55], as well as with fat accumulation [56]. The regulatory functions of *P. succinatutens* (a succinate-utilizing bacterium) [57] and *O. valericigenes* (a valerate-producing bacterium) [58] in host metabolism are poorly understood. Our data indicated that the three core-predominant gut microbes play important roles in maintaining host organ indexes (including the heart, spleen, and thymus). The Human Microbiome Project has also raised some fundamental scientific questions regarding functional contributions of gut microbiota to the host and has suggested potential relationships between the gut microbiota and organ indexes [59]. Our findings revealed that the three core-predominant gut microbes play important roles in promoting intestinal epithelial barrier function, thus suggesting the potential beneficial functions of gut microbes in maintaining host intestinal health [60]. Our data also showed that the three core-predominant gut microbes significantly increased the crypt depths in the duodenum and ileum, suggesting the regulatory functions of gut microbiota in maintaining gut mucosal morphology, as shown previously [10]. Thus, our results have linked the three core gut microbes to host organ indexes and intestinal mucosal morphology, improving our understanding regarding the contribution of core gut microbial functions to host physiology and growth.

The gut microbiota play important roles in the host serum metabolome [53, 61, 62], and gut microbiota-derived metabolites are involved in host metabolism [63]. Several studies have also revealed the critical roles of the gut microbiota–brain, gut microbiota–liver, and gut microbiota–kidney axes in host health [64–66]. Our results demonstrated that the three core-predominant gut microbes significantly altered the profiles of serum and fecal metabolites, thus providing insights regarding gut microbiota-mediated host metabolism. Seven common metabolic pathways, including “steroid hormone biosynthesis,” “primary bile acid biosynthesis,” “phenylalanine, tyrosine and tryptophan biosynthesis,” “phenylalanine metabolism,” “glycerolipid metabolism,” “cholesterol metabolism,” and “bile secretion,” were significantly enriched with those differentially serum metabolites induced by the three core-predominant gut microbes, respectively. Moreover, five common metabolic pathways, including “vitamin digestion and absorption,” “vitamin B6 metabolism,” “tryptophan metabolism,” “phenylalanine metabolism,” and “metabolic pathways,” were significantly enriched with those differentially fecal metabolites induced by the three core-predominant gut microbes, respectively. Previous studies have also shown that the KEGG pathways enriched by the differential metabolites in serum and feces may differ as the locations of samples are distinct [67, 68]. These findings suggested that the alterations in host phenylalanine metabolism may be induced by the core gut microbes-mediated phenylalanine metabolism in the intestine. Previous studies have also indicated that some gut microbes are involved in the phenylalanine metabolism [69, 70]. Based on this, our results have established links between the core-predominant gut microbes and host nutrient metabolism, further improving our understanding of gut microbiota–host interaction.

In sum, we have systematically analyzed the structure and function of the pig gut microbiome using metagenomics and 16S rRNA gene sequencing. We established an expanded gut microbial reference catalog comprising 17 020 160 genes. Gut resistome analysis indicated a large risk of dissemination of antimicrobial-resistant gut microbes in commercial pigs. Furthermore, our data indicated that the three core-predominant gut microbes (including *P. succinatutens*, *P. copri*, and *O. valericigenes*) play important roles in maintaining host organ indexes, intestinal epithelial barrier function, intestinal mucosal morphology, and nutrition metabolism.

## Materials and methods

### Animals and samples collection

Fifty-six weaned piglets and 56 finishing pigs from seven breeds (commercial Duroc × [Landrace × Yorkshire], native Tibetan miniature, native Laiwu, native Shaziling, native Congjiang miniature, native Huanjiang miniature, and native Ningxiang pigs; *n* = 8) were used as described previously [28]. All the pigs were provided food and water ad libitum. Information on age, sex, and diet of pigs was presented in our previous study [28]. Fecal samples were collected from each pig and stored in liquid nitrogen. All experimental procedures for pigs were approved by the Institutional Animal Care and Use Committee of Huazhong Agricultural University (approval number: HZAUSW-2018-026).

### DNA extraction, metagenomic sequencing, and data analysis

DNA extraction [71] and metagenomics sequencing [67] were performed as described previously. Briefly, microbial genomic DNA from 112 fecal samples was released using bead-beating method and extracted using the cetyltrimethylammonium bromide method. The metagenome libraries were constructed through specific preparation kits (Illumina) and sequenced (2 × 150 paired-end) on a HiSeq X Ten System (Illumina). The raw data were filtered using SOAPnuke software (v1.5.6), and the host genomic reads were trimmed using Bowtie2 software (v2.2.5). Open reading frames were predicted using MetaGeneMark software (v3.26), and the predicted genes were clustered using CD-Hit software (v4.6.6). The previously reported NR genes in the pig gut microbiome [29] were then integrated into our de novo-assembled gene catalog. After removing redundant genes using the CD-Hit software with the same parameters, a new integrated NR gene catalog (including 17 020 160 NR genes) was constructed in the pig gut microbiome.

The NR gene abundance profile was constructed using the Salmon software (v0.9.1). Rarefaction curves of NR genes were drawn using R software (v3.1.1) by sampling 10 times. The functions of NR genes were annotated based on public databases (including NR, KEGG, and ARDB) by Diamond software, and alignment results with hit scores >60 and E values <1e-5 were retained. The PCA for KOs in animal gut microbiome (including human, pig, mouse, chicken, and monkey) was performed by package “ade4” from R software. The PCoA of KOs, ARGs, and NR genes was conducted by the package “vegan” of R software. Venn diagram for KOs was drawn by the package “VennDiagram” of R software. Histograms for taxonomic compositions were plotted by package “gplots” of R software.

The prokaryotic genome was reconstructed from metagenomic data using metaWRAP software (v1.1.5). Briefly, the metagenomic binning was performed based on the metagenomic assembly contigs and clean reads using the metaWRAP “binning” module that includes three binning software programs (CONCOCT, MaxBin2, and metaBAT2). Then, the MAGs in each sample were obtained using the “bin refinement” module. The CheckM software (v1.0.12) was used to evaluate contamination and completeness of the bins according to the quality evaluation criteria, and MAGs exhibiting completeness >50% and contamination <5% were retained. In total, 10 738 MAGs in 112 pig gut metagenomic samples were constructed, and dRep software (v2.5.4) was used to remove duplicate MAGs with 90% Mash average nucleotide identity (ANI) for the primary cluster and 99% ANI similarity values with at least 30% overlap for the secondary clusters of each primary cluster. The final 4910 NR MAGs were taxonomically classified, and marker genes were selected using the GTDB-tk software (v1.0.2). The FastTree software (v2.1.10) was used to construct phylogenetic trees, which were visualized using the iTOL software.

The metagenomic sequencing was used to investigate the relative abundance of three microbial species (*P. succinatutens, P. copri*, and *O. valericigenes*) in the feces of GF mice (FMT group in Fig. 6) that were orally gavaged a fecal suspension from commercial Duroc × [Landrace × Yorkshire] pigs every 3 days. Heatmap for relative abundances of three microbial species (*P. succinatutens, P. copri*, and *O. valericigenes*) was generated using package “gplots” of R software.

### Bacterial 16S rRNA gene amplicon sequencing and data analysis

The V4 region of the bacterial 16S rRNA gene was amplified using the following primers: F: 5′–GTGTGCCAGCMGCCGCGGTAA–3′ and R: 5′–GGACTACHVGGGTWTCTAAT–3′. It was then sequenced on a HiSeq 2500 System (Illumina) with PE250 strategy. The melting temperature was 56°C and 30 PCR cycles were performed. Clean data were obtained as described previously [71]. Clean reads with overlaps were merged into tags using PLASH software (v1.2.11). These tags were clustered into OTUs using USEARCH scripts (v7.0.1090) and taxonomically classified using the RDP Classifier (v2.14). The Chao index was calculated using Mothur software (v1.31.2). The rarefaction curves were drawn using the function “plot” of R software. PCA of OTUs was performed by the package “ade4” of R software. The histograms and heatmaps for taxonomic compositions were drawn by the package “gplots” of R software. Beta diversity analysis was performed by QIIME software (v1.80), and scatter plots from PCoA based on UniFrac distance and cluster trees were drawn by package “vegan” of R software.

16S rRNA gene sequencing was performed to investigate relative abundance of *O. valericigenes* in the feces of SPF mice (SPF group in Fig. 6). The 16S rRNA gene sequencing and analysis of eight fecal samples from SPF mice were performed as described previously [67]. Heatmap for the relative abundances of the *O. valericigenes* was generated by the package “gplots” of R software.

### Culture of bacterial strains

Bacterial strains (*P. succinatutens, P. copri*, and *O. valericigenes*) were isolated from fresh pig feces in an anaerobic incubator. *P. succinatutens* was cultured in modified GAM medium supplemented with 1% succinic acid at 37°C as mentioned previously [57]. *P. copri* was cultured in a modified medium (containing 5 g xylan, 20 g tryptone, 5 g yeast extract, 5 g NaCl, 0.05 g K_2_HPO_4,_ 0.05 g KH_2_PO4, 0.3 g L-cysteine hydrochloride, 0.005 g hemin, and 0.001 g vitamin K_1_ per liter) at 37°C. *O. valericigenes* was cultured in modified GYP medium supplemented with 1% α-lactose at 37°C as described previously [58]. In the microbial isolation experiments, both vancomycin (final concentration: 20 mg/l) and fluconazole (final concentration: 25 mg/l) were added to the medium. These strains were identified based on the results of 16S rRNA gene sequencing.

### Oral gavage of bacterial strains and FMT in GF mice

Fourty-six GF Kunming mice (three weeks of age) and eight SPF Kunming mice (3 weeks of age) of similar weights were used to evaluate the roles of gut core-predominant microbes and FMT in host physiology. The GF mice were randomly assigned into five groups and orally gavaged 150 μl sterile phosphate buffered saline (PBS) (*n* = 10), *P. succinatutens* suspension (*n* = 10), *P. copri* suspension (*n* = 11), *O. valericigenes* suspension (*n* = 8), and fecal suspension of commercial Duroc × [Landrace × Yorkshire] pigs (*n* = 7), respectively, every 3 days. The concentration of bacterial suspension used was 10^8^ CFU/ml. The fecal suspension was prepared as mentioned previously [12]. SPF mice were orally gavaged with sterile PBS (*n* = 8) every 3 days. Experiments were performed when the mice were 3–8 weeks of age. All the mice consumed the same food and drank the same water ad libitum. All experimental procedures for mice were approved by the Institutional Animal Care and Use Committee of Huazhong Agricultural University (approval number: HZAUMO-2019-087).

### Measurements of organ indexes, blood routine indexes, serum immunoglobulins levels, and serum diamine oxidase activity

After the mice were euthanized, their organs (heart, liver, spleen, kidney, and thymus) and epididymal fat were weighed immediately. Organ index was calculated as the ratio of organ to body weight. Epididymal fat index was calculated as the ratio of epididymal fat to the body weight. Routine blood indexes were examined using an automatic hematology analyzer (BC-2800vet, Mindray). The levels of serum immunoglobulins (IgA, IgG, and IgM) and IFN-γ were examined using the enzyme-linked immunosorbent assay kits (H108-1, H106, H109, and H025, Nanjing Jiancheng Bioengineering Institute [NJBI], China). Serum diamine oxidase activity was measured using an assay kit (NJBI, A088).

### Analyses of intestinal mucosal morphology

Hematoxylin and eosin staining was used to assess intestinal mucosal morphology according to a previously described procedure [72]. Villi heights, villus width, and crypts depths in the duodenum, jejunum, and ileum were measured individually. Representative images of the intestinal tissues at 40×, 100×, and 200× magnifications were obtained using a light microscope.

### Identification of metabolites in serum and feces using metabolomics

Global metabolite profiling of serum and feces was performed using ultra-performance liquid chromatography combined with mass spectrometry as mentioned previously [67], and the procedures have been described in detail [67]. The differential metabolites were identified according to the criteria of variable importance in projection (VIP) ≥ 1, absolute Log_2_ (fold change) ≥ 1, and *P* value <0.05. A Venn diagram of differential metabolites was created using OmicShare. The OPLS-DA plot, volcano plot, and bubble diagram were created by R software.

### Complete genome sequencing of bacterial strains

Bacterial strains (*P. succinatutens*, *P. copri*, and *O. valericigenes*) were cultured in those media as described above, respectively. Bacterial genomic DNA was extracted using a kit (TIANGEN, DP302). The genomes of these strains were sequenced using the PacBio RS II System (Pacific Biosciences) and HiSeq 4000 System (Illumina). Complete chromosome-level genomic contigs were assembled using Celera Assembler against a high-quality corrected circular consensus sequence subreads set. The genome analysis toolkit and short oligonucleotide alignment program (SOAP) tool packages (including SOAP2, SOAPsnp, and SOAPindel) were used to make single-base corrections to improve genome sequences accuracy. Gene prediction was performed by the Glimmer 3 software. The KEGG database was used for annotation of general function.

### Western blot analysis

Whole cell lysates of intestinal mucosa from approximately the middle positions in intestinal tracts (duodenum, jejunum, and ileum) were prepared in RIPA lysis buffer (Sangon Biotech, C500005), as described previously [72–75]. Western blotting was performed as described previously [72]. Primary antibodies included anti-ZO-1 (ABclonal, A11417), anti-E-cadherin (Cell Signaling Technology, 14472S), anti-Connexin 43 (Proteintech, 26 980–1-AP), and anti-β-tubulin (Proteintech, 66 240–1-Ig). HRP-conjugated secondary antibodies (Proteintech, SA00001–1, and SA00001–2) were purchased from the Proteintech (China).

### Statistical analysis

The GraphPad Prism (version 6.0c) and R (v3.1.1) software were used. Detailed descriptions of the statistical methods are provided in figure legends. Differences were considered significant when the *P* value was <0.05.

## Author contributions

Jun Hu and Xianghua Yan designed the study. Jun Hu and Qiliang Hou performed the experiments. Jun Hu, Jianwei Chen, and Xiangru Wang analyzed the data. Libao Ma, Yong Zhang, Xiangfeng Kong, Xingguo Huang, Zhonglin Tang, and Hong Wei contributed to collection of experimental samples. Jun Hu, Xiangru Wang, and Xianghua Yan wrote the manuscript. All authors have reviewed and approved the final version of the manuscript.

## Conflicts of interest

None declared.

## Funding

This work was supported by the National Natural Science Foundation of China (31925037 and 32230099), Hubei Hongshan Laboratory (2021hszd018), and Fundamental Research Funds for the Central Universities (Project 2662023DKPY002) to X.Y., the National Natural Science Foundation of China (32102499), the National Postdoctoral Program for Innovative Talents of China (BX20190133), the Postdoctoral Science Foundation of China (2019 M662671), the Natural Science Foundation of Hubei Province (2022CFB358 and 2021CFA018), and the Hubei Provincial Postdoctoral Innovative Post Project of China to J.H.

## Data availability

Raw sequencing data were deposited in the China National GeneBank Sequence Archive (CNSA) of the China National GeneBank Database (CNGBdb) under the accession number CNP0002106. The pig gut microbial gene catalog and MAGs were deposited in the Figshare database (link: https://figshare.com/s/dcd3af1ad5c5717bc12c; DOI: 10.6084/m9.figshare.24481501). The data for complete genomes of three bacterial strains were deposited in the Figshare database (link: https://figshare.com/s/41f6bc2ec64e6674e955; DOI: 10.6084/m9.figshare.24486163).

.

## Supplementary Material

Supplementary_Figure_1_wrad037

Supplementary_Figure_2_wrad037

Supplementary_Figure_3_wrad037

Supplementary_Figure_4_wrad037

Supplementary_Figure_5_wrad037

Supplementary_Figure_6_wrad037

Supplementary_Figure_7_wrad037

Supplementary_Figure_8_wrad037

Supplementary_Figure_9_wrad037

Clean_revised_supplementary_figure_legends

## References

[1] Gresse R , Chaucheyras-Durand F, Fleury MA et al. Gut microbiota dysbiosis in postweaning piglets: understanding the keys to health. Trends Microbio*l* 2017;25:851–73. 10.1016/j.tim.2017.05.004. 28602521

[2] Maxmen A . Model pigs face messy path. Natur*e* 2012;486:453. 10.1038/486453a. 22739291

[3] Meurens F , Summerfield A, Nauwynck H et al. The pig: a model for human infectious diseases. Trends Microbio*l* 2012;20:50–7. 10.1016/j.tim.2011.11.002. 22153753 PMC7173122

[4] Heinritz SN , Mosenthin R, Weiss E. Use of pigs as a potential model for research into dietary modulation of the human gut microbiota. Nutr Res Re*v* 2013;26:191–209. 10.1017/S0954422413000152. 24134811

[5] Koh A , De Vadder F, Kovatcheva-Datchary P et al. From dietary fiber to host physiology: short-chain fatty acids as key bacterial metabolites. Cel*l* 2016;165:1332–45. 10.1016/j.cell.2016.05.041. 27259147

[6] Magnusdottir S , Ravcheev D, de Crecy-Lagard V et al. Systematic genome assessment of B-vitamin biosynthesis suggests co-operation among gut microbes. Front Gene*t* 2015;6:148. 10.3389/fgene.2015.00148. 25941533 PMC4403557

[7] Pull SL , Doherty JM, Mills JC et al. Activated macrophages are an adaptive element of the colonic epithelial progenitor niche necessary for regenerative responses to injury. Proc Natl Acad Sci U S *A* 2005;102:99–104. 10.1073/pnas.0405979102. 15615857 PMC544052

[8] Buffie CG , Pamer EG. Microbiota-mediated colonization resistance against intestinal pathogens. Nat Rev Immuno*l* 2013;13:790–801. 10.1038/nri3535. 24096337 PMC4194195

[9] Spencer SP , Fragiadakis GK, Sonnenburg JL. Pursuing human-relevant gut microbiota-immune interactions. Immunit*y* 2019;51:225–39. 10.1016/j.immuni.2019.08.002. 31433970 PMC7205593

[10] Sommer F , Backhed F. The gut microbiota-masters of host development and physiology. Nat Rev Microbio*l* 2013;11:227–38. 10.1038/nrmicro2974. 23435359

[11] Lemon KP , Armitage GC, Relman DA et al. Microbiota-targeted therapies: an ecological perspective. Sci Transl Me*d* 2012;4:137rv5. 10.1126/scitranslmed.3004183. PMC5725196 22674555

[12] Hu J , Ma LB, Nie YF et al. A microbiota-derived bacteriocin targets the host to confer diarrhea resistance in early-weaned piglets. Cell Host Microb*e* 2018;24:817–832.e8. 10.1016/j.chom.2018.11.006. 30543777

[13] Hu LS , Geng SJ, Li Y et al. Exogenous fecal microbiota transplantation from local adult pigs to crossbred newborn piglets. Front Microbio*l* 2018;8:2663. 10.3389/fmicb.2017.02663. 29375527 PMC5767267

[14] Wang ZX , He YZ, Wang CD et al. Variations in microbial diversity and metabolite profiles of female landrace finishing pigs with distinct feed efficiency. Front Vet Sc*i* 2021;8:702931. 10.3389/fvets.2021.702931. 34307537 PMC8299115

[15] Jiang H , Fang SM, Yang H et al. Identification of the relationship between the gut microbiome and feed efficiency in a commercial pig cohort. J Anim Sc*i* 2021;99:skab045. 10.1093/jas/skab045. 33570553 PMC7947963

[16] Gardiner GE , Metzler-Zebeli BU, Lawlor PG. Impact of intestinal microbiota on growth and feed efficiency in pigs: a review. Microorganism*s* 2020;8:1886. 10.3390/microorganisms8121886. 33260665 PMC7761281

[17] Bergamaschi M , Tiezzi F, Howard J et al. Gut microbiome composition differences among breeds impact feed efficiency in swine. Microbiome*.* 2020;8:110. 10.1186/s40168-020-00888-9. 32698902 PMC7376719

[18] Yang H , Xiang Y, Robinson K et al. Gut microbiota is a major contributor to adiposity in pigs. Front Microbio*l* 2018;9:3045. 10.3389/fmicb.2018.03045. 30619136 PMC6296290

[19] Wu CF , Lyu WT, Hong QH et al. Gut microbiota influence lipid metabolism of skeletal muscle in pigs. Front Nut*r* 2021;8:675445. 10.3389/fnut.2021.675445. 33928112 PMC8076524

[20] Pi Y , Wu YJ, Zhang XY et al. Gut microbiota-derived ursodeoxycholic acid alleviates low birth weight-induced colonic inflammation by enhancing M2 macrophage polarization. Microbiome*.* 2023;11:19. 10.1186/s40168-022-01458-x. 36721210 PMC9887892

[21] Wu JM , Wang JP, Lin ZS et al. *Clostridium butyricum* alleviates weaned stress of piglets by improving intestinal immune function and gut microbiota. Food Che*m* 2023;405:135014. 10.1016/j.foodchem.2022.135014. 36442249

[22] Zhang QQ , Vasquez R, Yoo JM et al. Dietary supplementation of *Limosilactobacillus mucosae* LM1 enhances immune functions and modulates gut microbiota without affecting the growth performance of growing pigs. Front Vet Sc*i* 2022;9:918114. 10.3389/fvets.2022.918114. 35847647 PMC9280434

[23] Ramayo-Caldas Y , Mach N, Lepage P et al. Phylogenetic network analysis applied to pig gut microbiota identifies an ecosystem structure linked with growth traits. ISME *J* 2016;10:2973–7. 10.1038/ismej.2016.77. 27177190 PMC5148198

[24] Levin D , Raab N, Pinto Y et al. Diversity and functional landscapes in the microbiota of animals in the wild. Scienc*e* 2021;372:eabb5352. 10.1126/science.abb5352. 33766942

[25] Turnbaugh PJ , Gordon JI. The core gut microbiome, energy balance and obesity. J Physio*l* 2009;587:4153–8. 10.1113/jphysiol.2009.174136. 19491241 PMC2754355

[26] Turnbaugh PJ , Hamady M, Yatsunenko T et al. A core gut microbiome in obese and lean twins. Natur*e* 2009;457:480–4. 10.1038/nature07540. 19043404 PMC2677729

[27] Neu AT , Allen EE, Roy K. Defining and quantifying the core microbiome: challenges and prospects. Proc Natl Acad Sci U S *A* 2021;118:e2104429118. 10.1073/pnas.2104429118. 34862327 PMC8713806

[28] Hu J , Chen JW, Hou QL et al. Core-predominant gut fungus *Kazachstania slooffiae* promotes intestinal epithelial glycolysis via lysine desuccinylation in pigs. Microbiome*.* 2023;11:31. 10.1186/s40168-023-01468-3. 36814349 PMC9948344

[29] Xiao L , Estelle J, Kiilerich P et al. A reference gene catalogue of the pig gut microbiome. Nat Microbio*l* 2016;1:16161. 10.1038/nmicrobiol.2016.161. 27643971

[30] Chen CY , Zhou YY, Fu H et al. Expanded catalog of microbial genes and metagenome-assembled genomes from the pig gut microbiome. Nat Commu*n* 2021;12:1106. 10.1038/s41467-021-21295-0. 33597514 PMC7889623

[31] Yang H , Wu JY, Huang XC et al. ABO genotype alters the gut microbiota by regulating GalNAc levels in pigs. Natur*e* 2022;606:358–67. 10.1038/s41586-022-04769-z. 35477154 PMC9157047

[32] Wang XF , Tsai T, Deng FL et al. Longitudinal investigation of the swine gut microbiome from birth to market reveals stage and growth performance associated bacteria. Microbiom*e* 2019;7:109. 10.1186/s40168-019-0721-7. 31362781 PMC6664762

[33] Hou QC , Zhao FY, Liu WJ et al. Probiotic-directed modulation of gut microbiota is basal microbiome dependent. Gut Microbe*s* 2020;12:1736974. 10.1080/19490976.2020.1736974. 32200683 PMC7524168

[34] Fehlner-Peach H , Magnabosco C, Raghavan V et al. Distinct polysaccharide utilization profiles of human intestinal *Prevotella copri* isolates. Cell Host Microb*e* 2019;26:680–690.e5. 10.1016/j.chom.2019.10.013. 31726030 PMC7039456

[35] Li JH , Jia HJ, Cai XH et al. An integrated catalog of reference genes in the human gut microbiome. Nat Biotechno*l* 2014;32:834–41. 10.1038/nbt.2942. 24997786

[36] Xiao L , Feng Q, Liang SS et al. A catalog of the mouse gut metagenome. Nat Biotechno*l* 2015;33:1103–8. 10.1038/nbt.3353. 26414350

[37] Huang P , Zhang Y, Xiao KP et al. The chicken gut metagenome and the modulatory effects of plant-derived benzylisoquinoline alkaloids. Microbiom*e* 2018;6:211. 10.1186/s40168-018-0590-5. 30482240 PMC6260706

[38] Li XP , Liang SS, Xia ZK et al. Establishment of a *Macaca fascicularis* gut microbiome gene catalog and comparison with the human, pig, and mouse gut microbiomes. Gigascienc*e* 2018;7:giy100. 10.1093/gigascience/giy100. 30137359 PMC6137240

[39] Allen HK , Trachsel J, Looft T et al. Finding alternatives to antibiotics. Ann N Y Acad Sc*i* 2014;1323:91–100. 10.1111/nyas.12468. 24953233

[40] Chen W , Fang GF, Wang SD et al. Longissimus lumborum muscle transcriptome analysis of Laiwu and Yorkshire pigs differing in intramuscular fat content. Genes Geno*m* 2017;39:759–66. 10.1007/s13258-017-0540-9.

[41] Wang H , Wang J, Yang DD et al. Expression of lipid metabolism genes provides new insights into intramuscular fat deposition in Laiwu pigs. Asian Austral J Ani*m* 2020;33:390–7. 10.5713/ajas.18.0225. PMC7054625 31480195

[42] Shang P , Zhang B, Zhang J et al. Expression and single-nucleotide polymorphisms of the H-FABP gene in pigs. Gen*e* 2019;710:156–60. 10.1016/j.gene.2019.05.061. 31173805

[43] Jia W , Xie GX, Jia WP. Bile acid-microbiota crosstalk in gastrointestinal inflammation and carcinogenesis. Nat Rev Gastro Hepa*t* 2018;15:111–28. 10.1038/nrgastro.2017.119. PMC5899973 29018272

[44] Cai J , Sun LL, Gonzalez FJ. Gut microbiota-derived bile acids in intestinal immunity, inflammation, and tumorigenesis. Cell Host Microb*e* 2022;30:289–300. 10.1016/j.chom.2022.02.004. 35271802 PMC8923532

[45] Jeong HS , Kim DW, Chun SY et al. Native pig and chicken breed database: NPCDB. Asian Austral J Ani*m* 2014;27:1394–8. 10.5713/ajas.2014.14059. PMC4150170 25178289

[46] Makki K , Deehan EC, Walter J et al. The impact of dietary fiber on gut microbiota in host health and disease. Cell Host Microb*e* 2018;23:705–15. 10.1016/j.chom.2018.05.012. 29902436

[47] Cheng PH , Wang Y, Liang J et al. Exploratory analysis of the microbiological potential for efficient utilization of fiber between Lantang and Duroc pigs. Front Microbio*l* 2018;9:1342. 10.3389/fmicb.2018.01342. 29988353 PMC6023970

[48] Casewell M , Friis C, Marco E et al. The European ban on growth-promoting antibiotics and emerging consequences for human and animal health. J Antimicrob Chemot*h* 2003;52:159–61. 10.1093/jac/dkg313. 12837737

[49] Zhou YY , Fu H, Yang H et al. Extensive metagenomic analysis of the porcine gut resistome to identify indicators reflecting antimicrobial resistance. Microbiome*.* 2022;10:39. 10.1186/s40168-022-01241-y. 35246246 PMC8895625

[50] Peng Z , Hu ZZ, Li ZG et al. Antimicrobial resistance and population genomics of multidrug-resistant *Escherichia coli* in pig farms in mainland China. Nat Commu*n* 2022;13:1116. 10.1038/s41467-022-28750-6. 35236849 PMC8891348

[51] Dimitrova L , Kaleva M, Zaharieva MM et al. Prevalence of antibiotic-resistant *Escherichia coli* isolated from swine faeces and lagoons in Bulgaria. Antibiotic*s* 2021;10:940. 10.3390/antibiotics10080940. 34438990 PMC8388900

[52] Ekhlas D , Sanjuan JMO, Manzanilla EG et al. Comparison of antimicrobial resistant *Escherichia coli* isolated from Irish commercial pig farms with and without zinc oxide and antimicrobial usage. Gut Patho*g* 2023;15:8. 10.1186/s13099-023-00534-3. 36829209 PMC9951511

[53] Pedersen HK , Gudmundsdottir V, Nielsen HB et al. Human gut microbes impact host serum metabolome and insulin sensitivity. Natur*e* 2016;535:376–81. 10.1038/nature18646. 27409811

[54] Kovatcheva-Datchary P , Nilsson A, Akrami R et al. Dietary fiber-induced improvement in glucose metabolism is associated with increased abundance of *Prevotella*. Cell Meta*b* 2015;22:971–82. 10.1016/j.cmet.2015.10.001. 26552345

[55] Alpizar-Rodriguez D , Lesker TR, Gronow A et al. *Prevotella copri* in individuals at risk for rheumatoid arthritis. Ann Rheum Di*s* 2019;78:590–3. 10.1136/annrheumdis-2018-214514. 30760471

[56] Chen CY , Fang SM, Wei H et al. *Prevotella copri* increases fat accumulation in pigs fed with formula diets. Microbiome*.* 2021;9:175. 10.1186/s40168-021-01110-0. 34419147 PMC8380364

[57] Watanabe Y , Nagai F, Morotomi M. Characterization of *Phascolarctobacterium succinatutens* sp nov., an asaccharolytic, succinate-utilizing bacterium isolated from human feces. Appl Environ Microbia*l* 2012;78:511–8. 10.1128/AEM.06035-11. PMC3255759 22081579

[58] Lino T , Mori K, Tanaka K et al. *Oscillibacter valericigenes* gen. nov., sp nov., a valerate-producing anaerobic bacterium isolated from the alimentary canal of a Japanese corbicula clam. Int J Syst Evol Microbia*l* 2007;57:1840–5. 10.1099/ijs.0.64717-0. 17684268

[59] Turnbaugh PJ , Ley RE, Hamady M et al. The human microbiome project. Natur*e* 2007;449:804–10. 10.1038/nature06244. 17943116 PMC3709439

[60] Ghosh S , Whitley CS, Haribabu B et al. Regulation of intestinal barrier function by microbial metabolites. Cell Mol Gastroenterol Hepato*l* 2021;11:1463–82. 10.1016/j.jcmgh.2021.02.007. 33610769 PMC8025057

[61] Chen LM , Zhernakova DV, Kurilshikov A et al. Influence of the microbiome, diet and genetics on inter-individual variation in the human plasma metabolome. Nat Me*d* 2022;28:2333–43. 10.1038/s41591-022-02014-8. 36216932 PMC9671809

[62] Greenhill C . Obesity: gut microbiome and serum metabolome changes. Nat Rev Endocrino*l* 2017;13:501. 10.1038/nrendo.2017.89. 28685768

[63] Krautkramer KA , Fan J, Backhed F. Gut microbial metabolites as multi-kingdom intermediates. Nat Rev Microbiol*.* 2021;19:77–94. 10.1038/s41579-020-0438-4. 32968241

[64] Morais LH , Schreiber HL, Mazmanian SK. The gut microbiota-brain axis in behaviour and brain disorders. Nat Rev Microbio*l* 2021;19:241–55. 10.1038/s41579-020-00460-0. 33093662

[65] Albillos A , de Gottardi A, Rescigno M. The gut-liver axis in liver disease: pathophysiological basis for therapy. J Hepato*l* 2020;72:558–77. 10.1016/j.jhep.2019.10.003. 31622696

[66] Giordano L , Mihaila SM, Eslami Amirabadi H et al. Microphysiological systems to recapitulate the gut-kidney axis. Trends Biotechno*l* 2021;39:811–23. 10.1016/j.tibtech.2020.12.001. 33419585

[67] Hu J , Chen JW, Xu XJ et al. Gut microbiota-derived 3-phenylpropionic acid promotes intestinal epithelial barrier function via AhR signaling. Microbiome*.* 2023;11:102. 10.1186/s40168-023-01551-9. 37158970 PMC10165798

[68] Liu SX , Liang SS, Liu H et al. Metabolite profiling of feces and serum in hemodialysis patients and the effect of medicinal charcoal tablets. Kidney Blood Press Re*s* 2018;43:755–67. 10.1159/000489912. 29804117

[69] Dodd D , Spitzer MH, Van Treuren W et al. A gut bacterial pathway metabolizes aromatic amino acids into nine circulating metabolites. Natur*e* 2017;551:648–52. 10.1038/nature24661. 29168502 PMC5850949

[70] Laursen MF , Sakanaka M, von Burg N et al. *Bifidobacterium* species associated with breastfeeding produce aromatic lactic acids in the infant gut. Nat Microbio*l* 2021;6:1367–82. 10.1038/s41564-021-00970-4. 34675385 PMC8556157

[71] Hu J , Nie YF, Chen JW et al. Gradual changes of gut microbiota in weaned miniature piglets. Front Microbio*l* 2016;7:1727. 10.3389/fmicb.2016.01727. 27853453 PMC5090779

[72] Hu J , Nie YF, Chen SF et al. Leucine reduces reactive oxygen species levels via an energy metabolism switch by activation of the mTOR-HIF-1 alpha pathway in porcine intestinal epithelial cells. Int J Biochem Cell *B* 2017;89:42–56. 10.1016/j.biocel.2017.05.026. 28583467

[73] Wang Y , Wu YP, Wang BK et al. Effects of probiotic *Bacillus* as a substitute for antibiotics on antioxidant capacity and intestinal autophagy of piglets. AMB Expres*s* 2017;7:52. 10.1186/s13568-017-0353-x. 28244029 PMC5328899

[74] Hu SL , Cao XF, Wu YP et al. Effects of probiotic *Bacillus* as an alternative of antibiotics on digestive enzymes activity and intestinal integrity of piglets. Front Microbio*l* 2018;9:2427. 10.3389/fmicb.2018.02427. 30405544 PMC6204369

[75] Wang Q , Zhan XL, Wang BK et al. Modified montmorillonite improved growth performance of broilers by modulating intestinal microbiota and enhancing intestinal barriers, anti-inflammatory response, and antioxidative capacity. Antioxidant*s* 2022;11:1799. 10.3390/antiox11091799. 36139873 PMC9495330

