# Supplementary figures and images for: Characterizing core microbiota and regulatory functions of the pig gut microbiome
Source: ISME J. 2024 Jan 12;18(1):wrad037. doi: 10.1093/ismejo/wrad037 (PMC10873858; doi:10.1093/ismejo/wrad037)

A

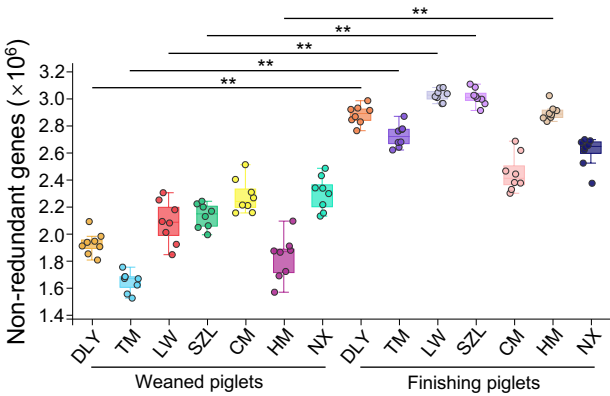

B

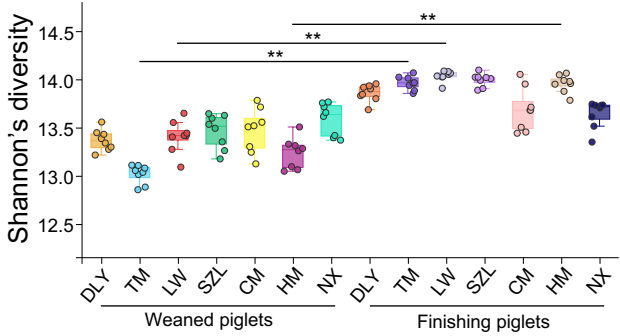

C

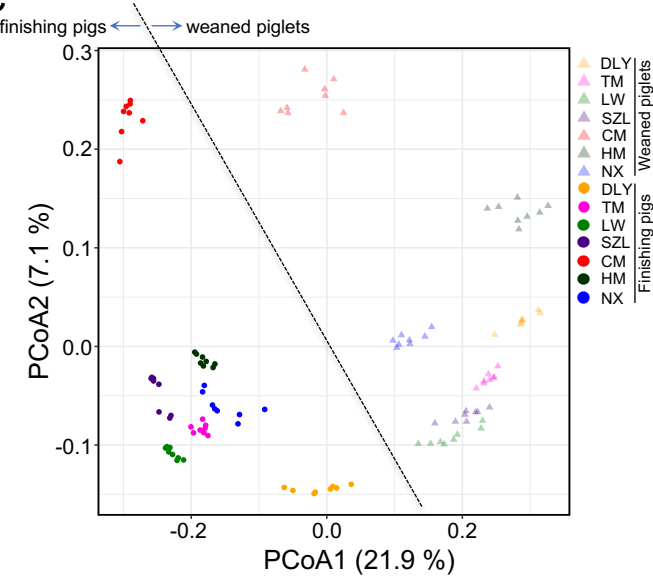

Supplement: Supplementary_Figure_1_wrad037 [file supplementary_figure_1_wrad037.pdf]

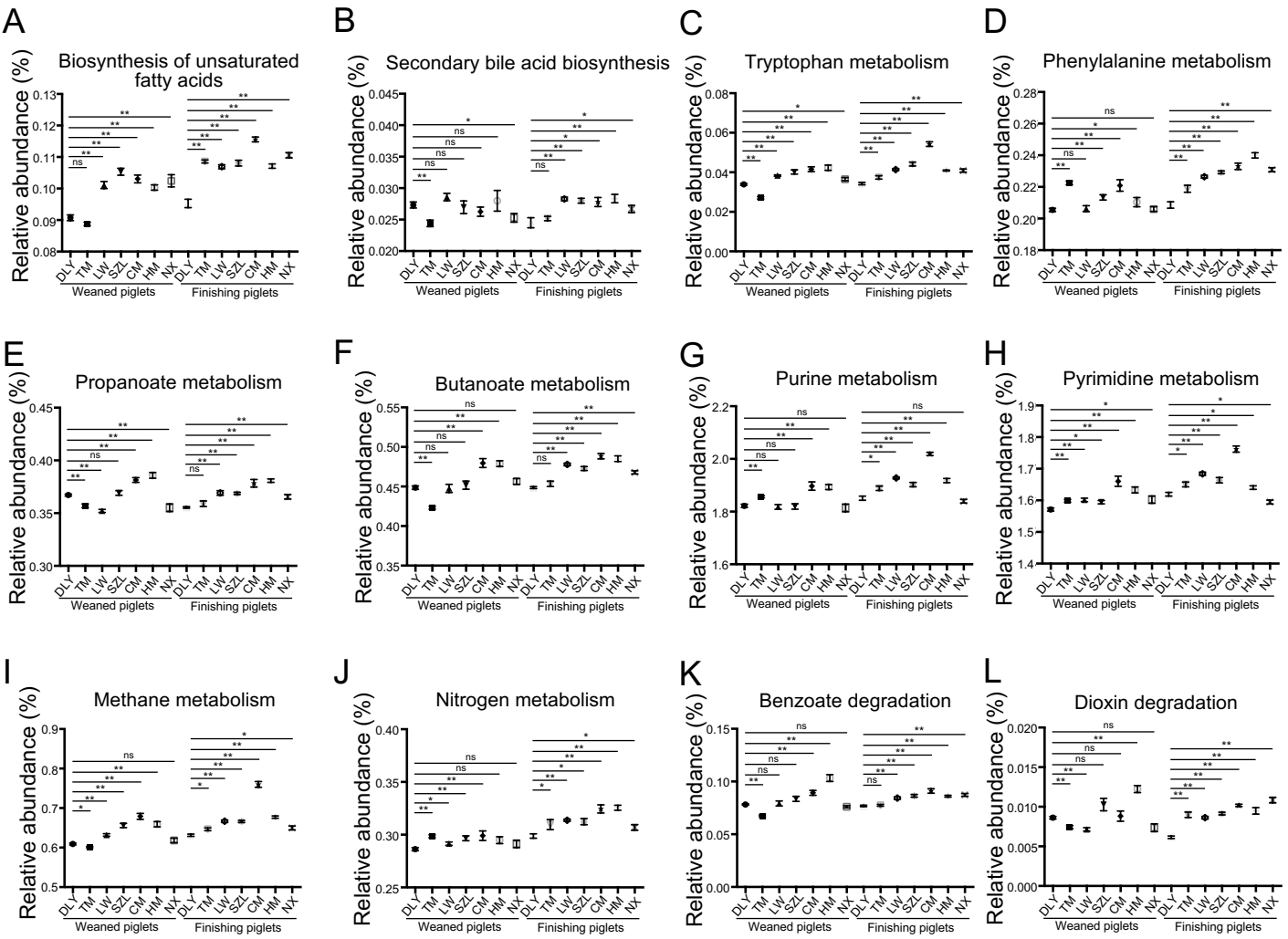

Supplement: Supplementary_Figure_2_wrad037 [file supplementary_figure_2_wrad037.pdf]

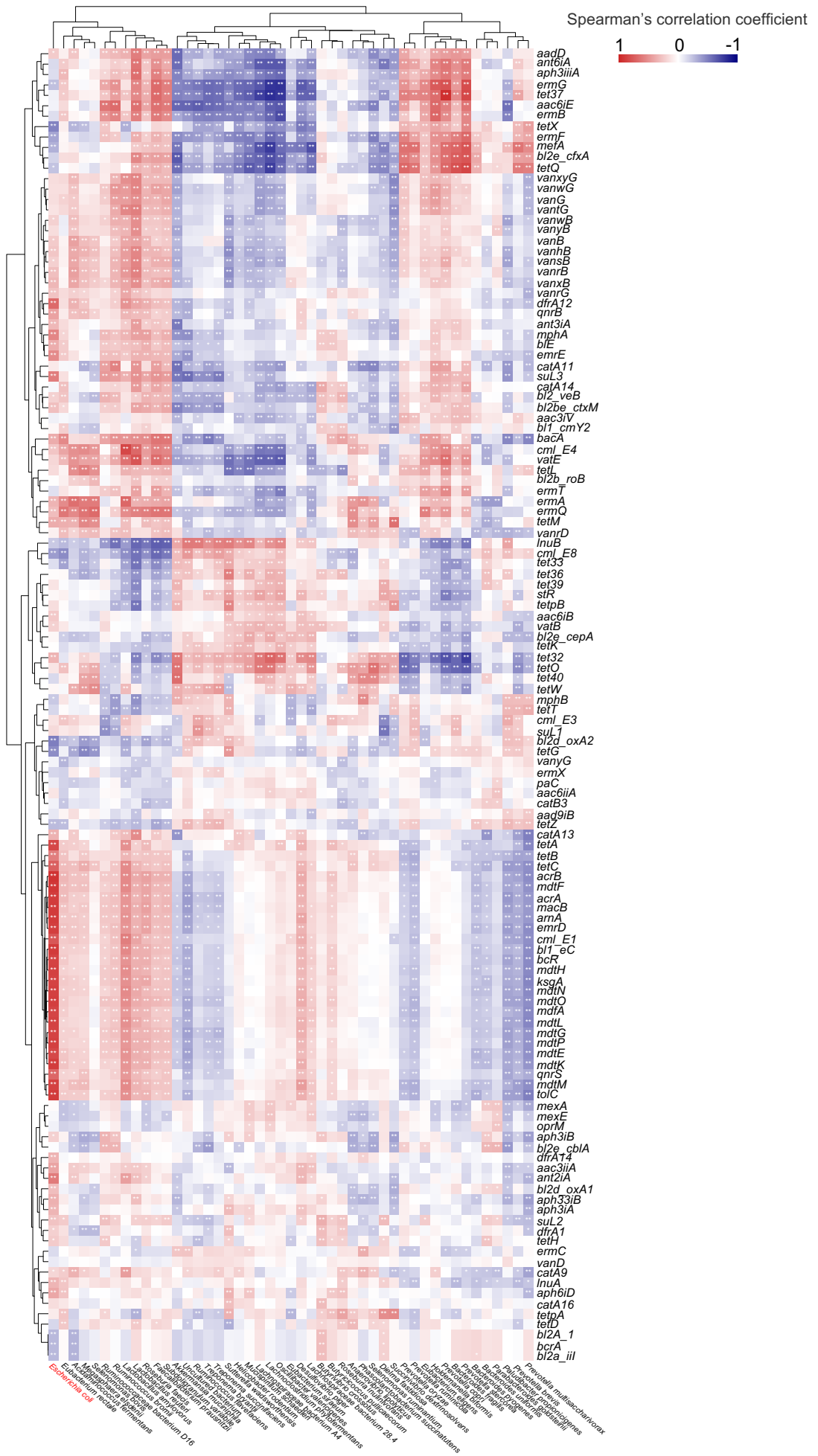

Supplement: Supplementary_Figure_3_wrad037 [file supplementary_figure_3_wrad037.pdf]

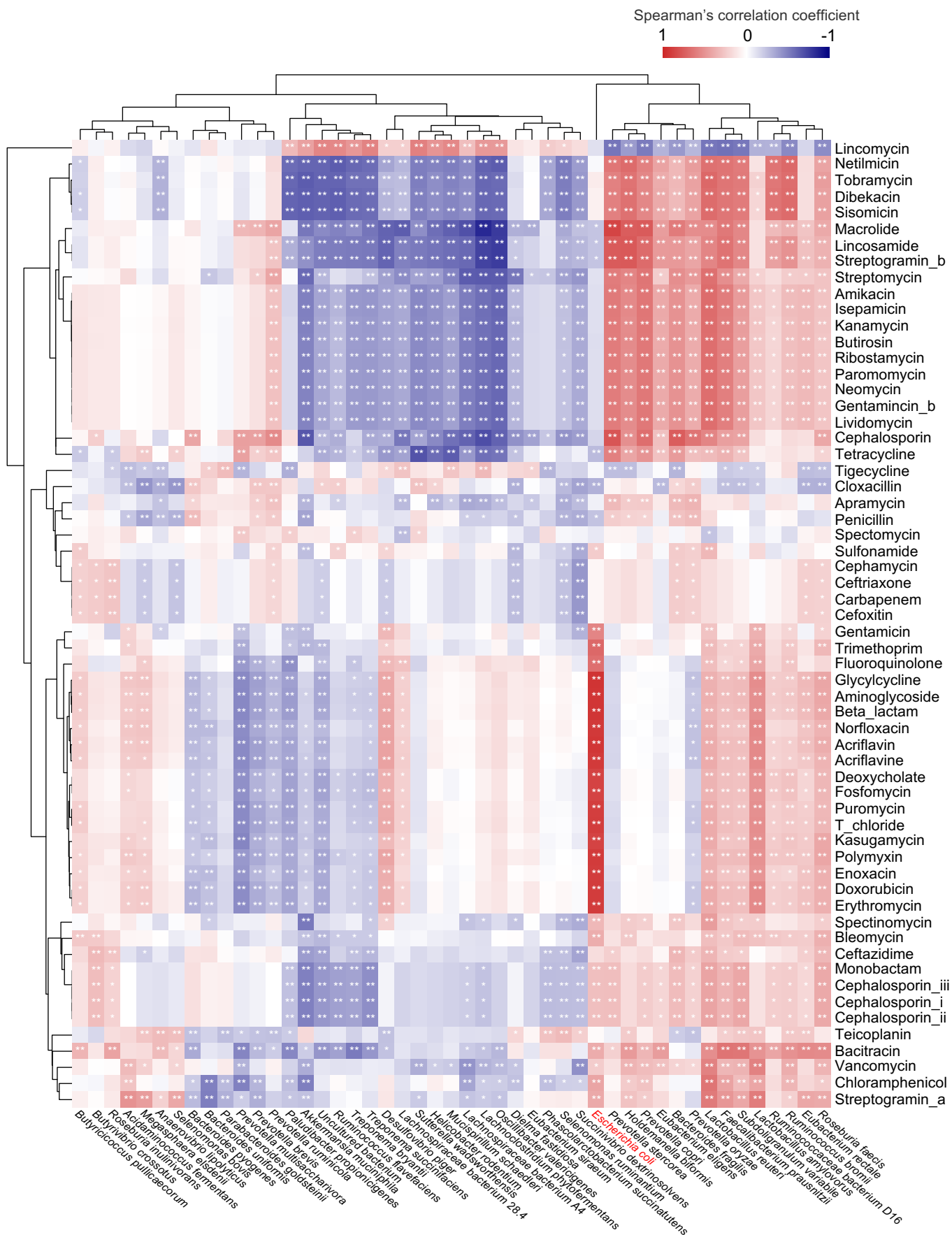

Supplement: Supplementary_Figure_4_wrad037 [file supplementary_figure_4_wrad037.pdf]

A

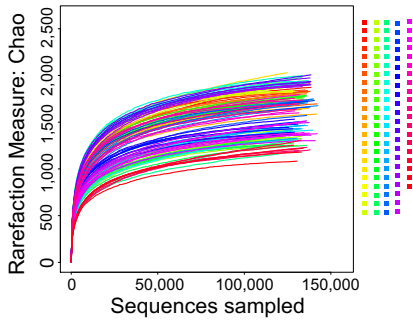

B

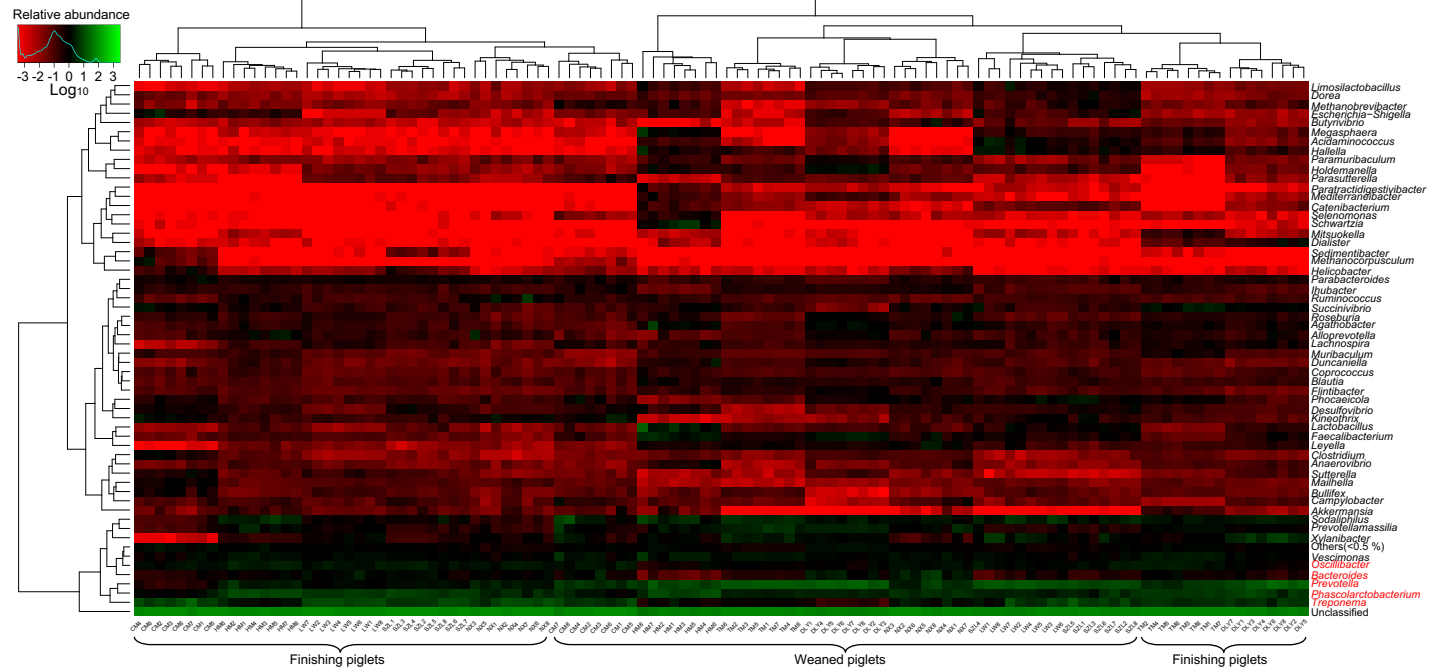

Supplement: Supplementary_Figure_5_wrad037 [file supplementary_figure_5_wrad037.pdf]

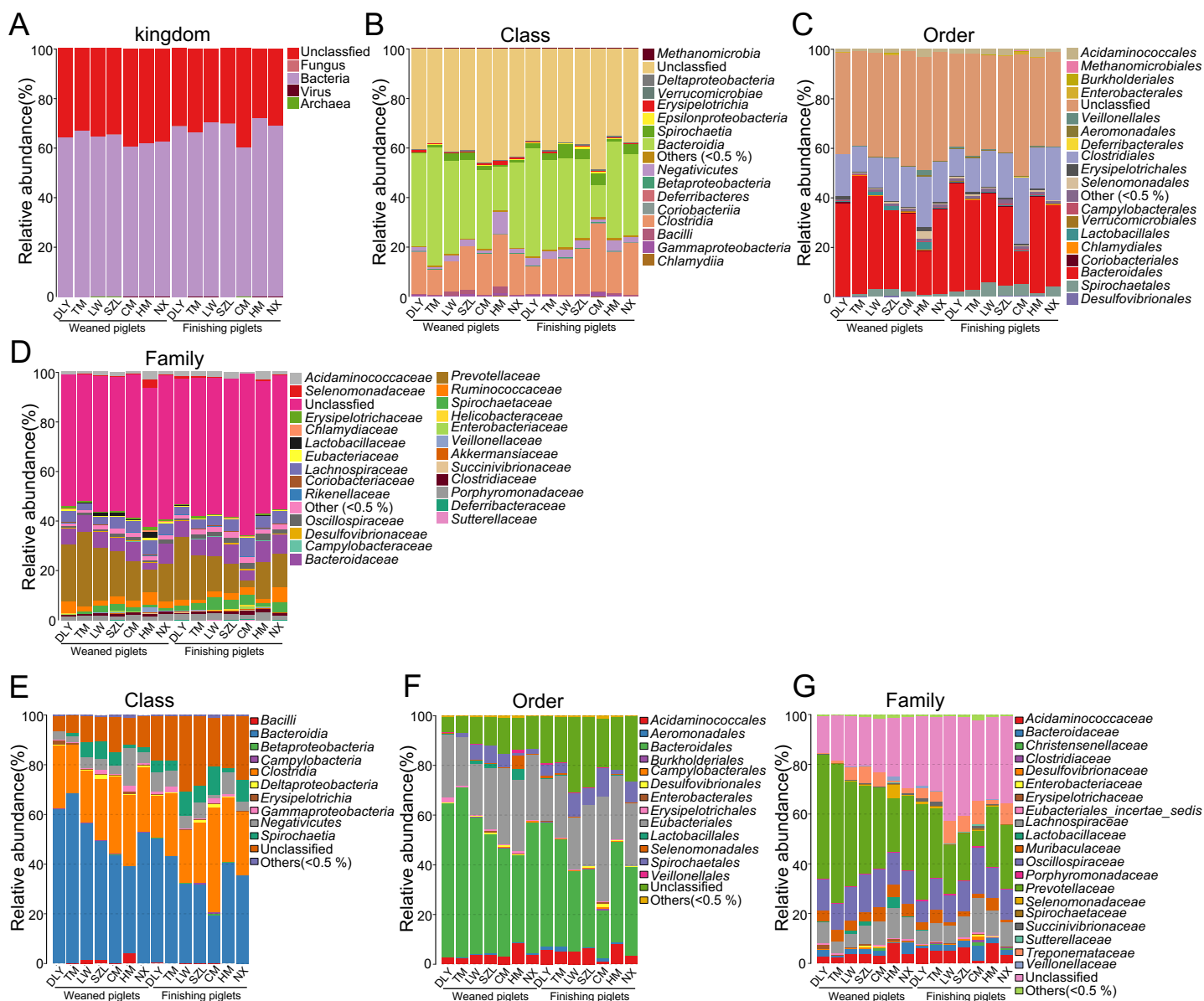

Supplement: Supplementary_Figure_6_wrad037 [file supplementary_figure_6_wrad037.pdf]

**A**

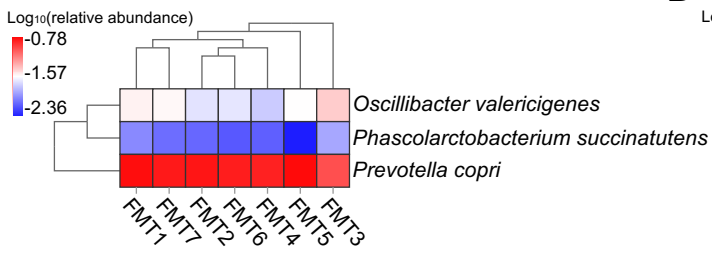

B

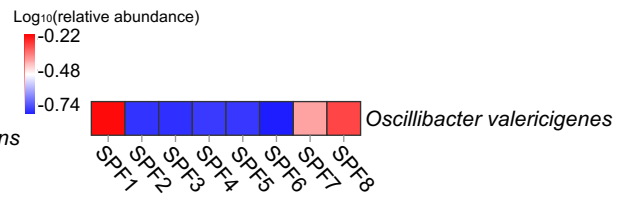

Supplement: Supplementary_Figure_7_wrad037 [file supplementary_figure_7_wrad037.pdf]

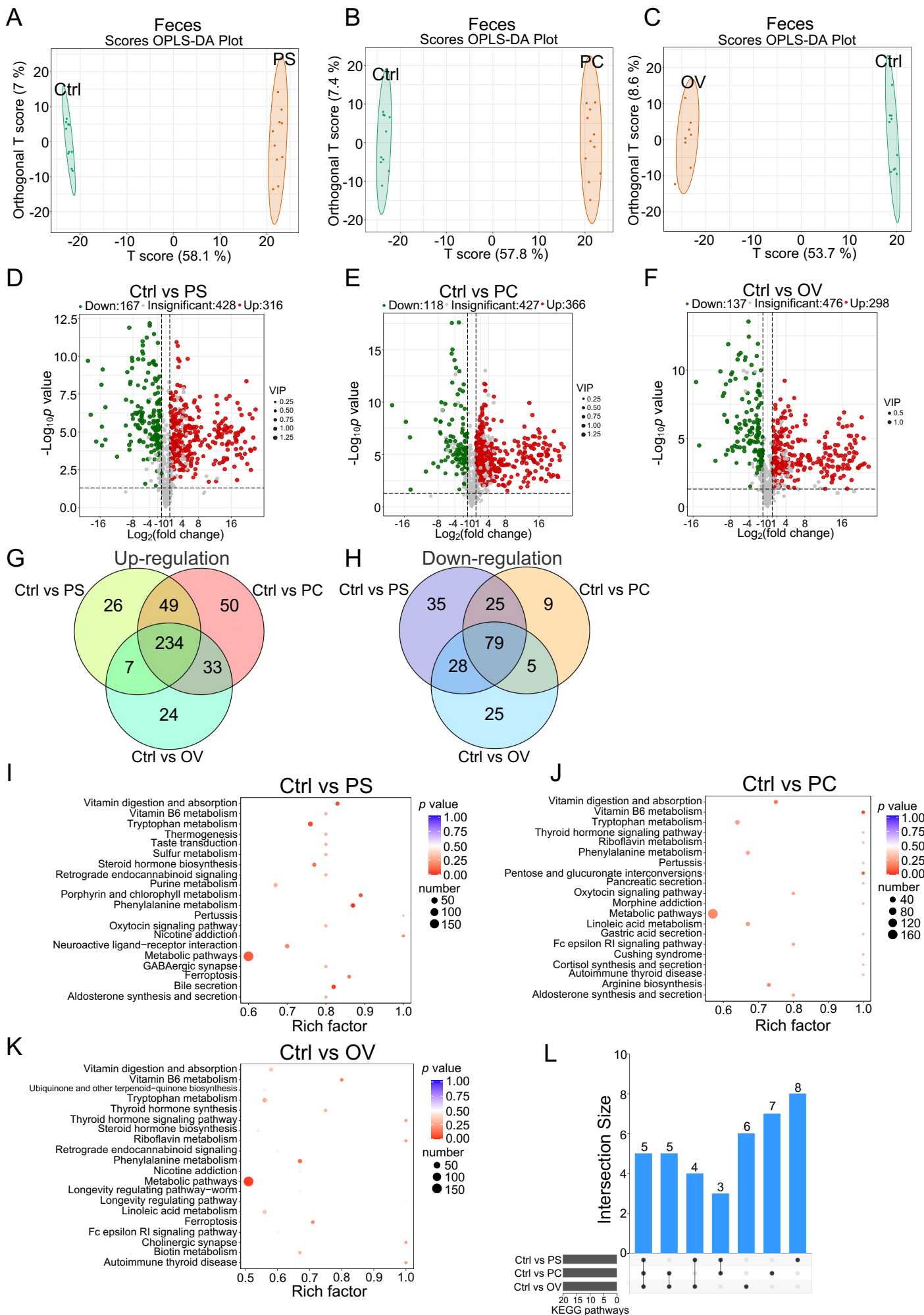

Supplement: Supplementary_Figure_8_wrad037 [file supplementary_figure_8_wrad037.pdf]

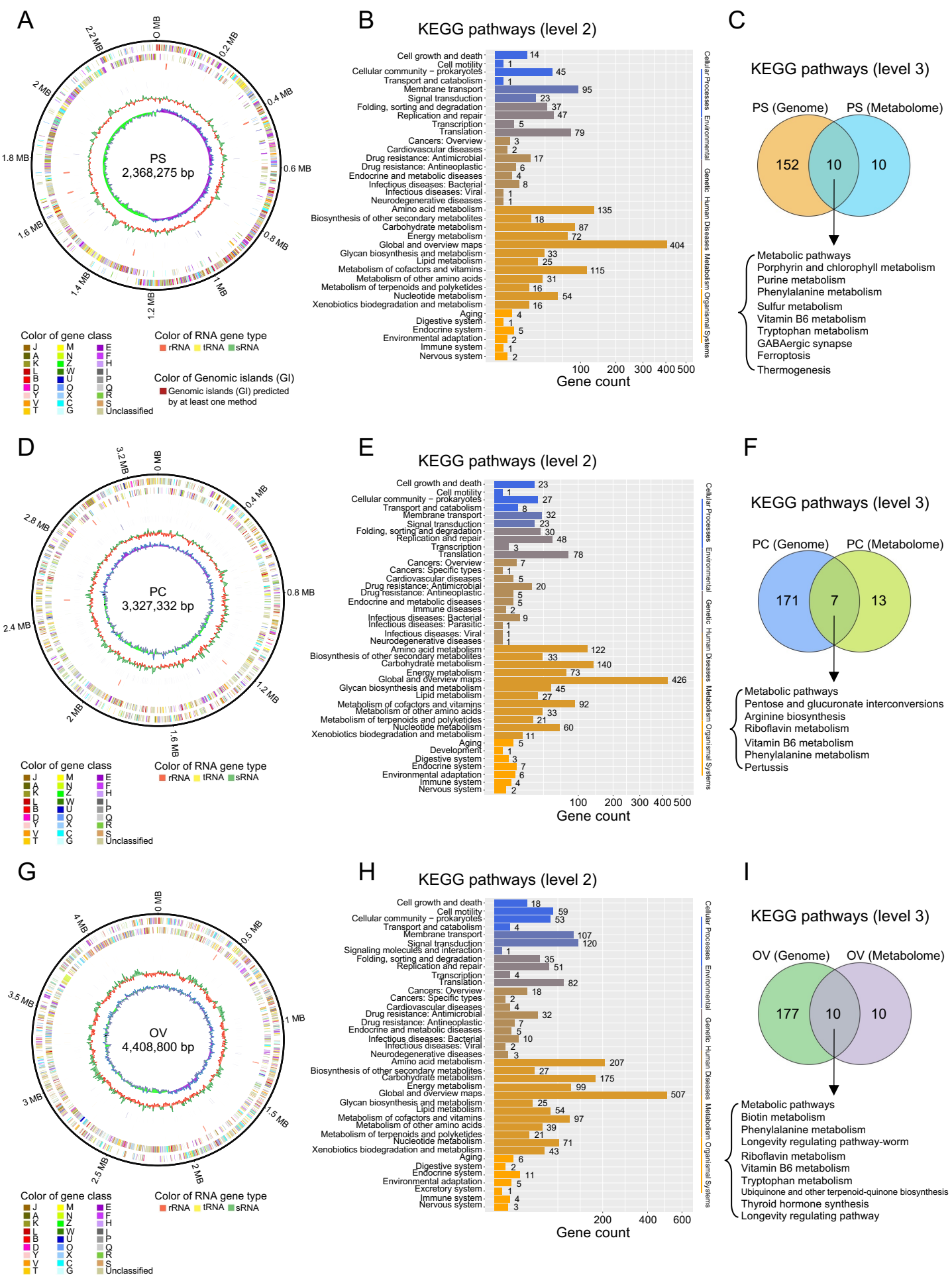

Supplement: Supplementary_Figure_9_wrad037 [file supplementary_figure_9_wrad037.pdf]
