# Supplementary material for: Characterizing core microbiota and regulatory functions of the pig gut microbiome
Source: ISME J. 2024 Jan 12;18(1):wrad037. doi: 10.1093/ismejo/wrad037 (PMC10873858; doi:10.1093/ismejo/wrad037)
Supplement: Clean_revised_supplementary_figure_legends [file clean_revised_supplementary_figure_legends.pdf]

# Supplementary Material Legends

**Fig. S1 Analyses of gut microbial NR gene numbers and diversity of gut microbial NR genes in pigs. A** The number of gut microbial NR genes quantified in 112 pigs from seven breeds. **B** Shannon indexes of gut microbial NR genes quantified in 112 pigs from seven breeds. **C** PCoA of gut microbial NR genes quantified in 112 pigs from seven breeds. Data are presented as mean ± SEM (n = 8) and were evaluated by the Kruskal-Wallis test. $^{**}p < 0.01$.

**Fig. S2 Comparative analyses of gut microbial KEGG pathways in pigs.** Comparative analyses of the relative abundances of microbial genes involved in KEGG pathways, including biosynthesis of unsaturated fatty acids (**A**), secondary bile acid biosynthesis (**B**), tryptophan metabolism (**C**), phenylalanine metabolism (**D**), propanoate metabolism (**E**), butanoate metabolism (**F**), purine metabolism (**G**), pyrimidine metabolism (**H**), methane metabolism (**I**), and nitrogen metabolism (**J**), benzoate degradation (**K**), and dioxin degradation (**L**), respectively. Data are presented as the mean ± SEM (n = 8). Significantly differentially abundant KEGG pathways were identified using the Kruskal-Wallis test. $^{**}p < 0.01$, $^{*}p < 0.05$; ns, not significant.

**Fig. S3 Correlation analysis of gut microbial species and antimicrobial resistance genes.** Heatmap for the Spearman's correlation analysis of microbial species (relative abundance >0.5 %) and antimicrobial resistance genes. $^{**}p < 0.01$, $^{*}p < 0.05$.

**Fig. S4 Correlation analysis of gut microbial species and antimicrobial resistance types.** Heatmap for the Spearman's correlation analysis of microbial species (relative abundance >0.5 %) and antimicrobial resistance types. $^{**}p < 0.01$, $^{*}p < 0.05$.

**Fig. S5 Rarefaction curves and heatmap analysis of gut bacterial community as revealed using 16S rRNA gene sequencing. A** Rarefaction curves for Chao index. **B** Heatmap analysis of bacterial genera.

**Fig. S6 Gut microbial taxonomic compositions in seven pig breeds. A-D** Gut microbial taxonomic compositions, including kingdom (A), class (B), order (C), and family (D), as revealed using metagenomics. **E-G** Gut bacterial taxonomic compositions, including class level (E), order level (F), and family level (G), as revealed using 16S rRNA gene sequencing.

**Fig. S7 Heatmap analysis of relative abundances of three core-predominant microbial species. A** Heatmap analysis of relative abundances of *P. succinatutens, P. copri*, and *O. valericigenes* in the feces of the mice (FMT group) revealed by metagenomics. **B** Heatmap analysis of relative abundances of *O. valericigenes* in the feces of the mice (SPF group) revealed using 16S rRNA gene sequencing.

**Fig. S8 Effects of core-predominant microbes on composition of fecal metabolites in germ-free mice. A-C** OPLS-DA for fecal metabolites in mice from groups (Ctrl and PS) (A), groups (Ctrl and PC) (B), groups (Ctrl and OV) (C) (Ctrl, control; PS, *P. succinatutens*; PC, *P. copri*; OV, *O. valericigenes*). **D-F** Volcano plot analysis of fecal metabolites in mice from groups (Ctrl and PS) (D), groups (Ctrl and PC) (E), and groups (Ctrl and OV) (F). **G** Venn diagram analysis of the fecal metabolites that are upregulated by the treatments with the three core gut microbes, respectively. **H** Venn diagram analysis of the fecal metabolites that are downregulated by the treatments with the three core gut microbes, respectively. **I-K** The KEGG enrichment analyses of the fecal metabolites altered by the treatments with *P. succinatutens* (I), *P. copri* (J), and *O. valericigenes* (K), respectively. **L** UpSet plot comparing the KEGG pathways enriched with the fecal differentially metabolites induced by the treatments with *P. succinatutens*, *P. copri*, and *O. valericigenes*, respectively. The differentially abundant metabolites were assessed based on VIP $\geq 1$ from the OPLS-DA, absolute Log2 (fold change) $\geq 1$, and $p$ value $< 0.05$.

**Fig. S9 Analyses of complete genome sequences of core-predominant microbes. A**

Circular representation of genome of *P. succinatutens* (PS, *P. succinatutens*). **B** KEGG pathway classification of *P. succinatutens* at level 2. **C** Venn diagram analysis of the KEGG pathways (level 3) annotated in *P. succinatutens* and the KEGG pathways (level 3) enriched by the fecal metabolites in GF mice that were orally administrated with *P. succinatutens*. **D** Circular representation of genome of *P. copri* (PC, *P. copri*). **E** KEGG pathway classification of *P. copri* at level 2. **F** Venn diagram analysis of the KEGG pathways (level 3) annotated in *P. copri* and the KEGG pathways (level 3) enriched by the fecal metabolites in GF mice that were orally administrated with *P. copri*. **G** Circular representation of genome of *O. valericigenes* (OV, *O. valericigenes*). **H** KEGG pathway classification of *O. valericigenes* at level 2. **I** Venn diagram analysis of the KEGG pathways (level 3) annotated in *O. valericigenes* and the KEGG pathways (level 3) enriched by the fecal metabolites in GF mice that were orally administrated with *O. valericigenes*.
